# Cerebellar Contributions to Cerebral Connectivity in Severe Mental Illness: A Developmental Cascade and Predictive-Modeling Framework with Autistic Disorder as the Focal Case

**DOI:** 10.3390/brainsci16090919

**Published:** 2026-08-28

**Authors:** Alan J. Lincoln

**Affiliations:** California School of Professional Psychology, Alliant University, San Diego, CA 92131, USA; alincoln@alliant.edu

**Keywords:** autism spectrum disorder, cerebellum, predictive error, connectivity, event-related potentials, neurodevelopment, genetic, severe mental illness, schizophrenia, psychosis spectrum

## Abstract

**Highlights:**

**What are the main findings?**

- Atypical patterns of brain growth and neuroconnectivity carry specific signatures within the heterogeneous forms of severe mental illness (SMI), a class treated here as encompassing autism, genetic syndromes, attention-deficit/hyperactivity disorder, and psychotic spectrum disorders.
- The cerebellum, particularly the neocerebellar vermis and cerebellar hemisphere areas VI and VII, may differentially affect early neurocognitive development.

**What are the implications of the main findings?**

- While the term “spectrum” may correctly capture expressed symptoms, both qualitatively and quantitatively, those may or may not have anything to do with common neuropathology or the neurodevelopmental course believed to be related to those symptoms.
- Methods of measuring neuropathology, neuroconnectivity, neurophysiology, and neurodevelopment can become confounded with the diagnostic system employed to characterize cohorts of those with SMI.

**Abstract:**

Severe mental illness (SMI) includes neuropsychiatric disorders of adult onset, but also those that begin to show symptoms in adolescence and early childhood. This review treats the psychotic spectrum disorders and autism as neurodevelopmental in origin, each arising from perturbation of early brain development rather than from a process beginning at the age of clinical presentation. Grouping them is a claim about developmental origin and not about shared pathogenesis: they differ in the nature and timing of the perturbation and in the cortical systems being organized when it occurs. The diversity of social, language, behavioral, and cognitive symptoms and traits observed within this class is now well recognized. The language adopted to recognize such diversity has employed the term “spectrum” (e.g., autism spectrum disorder (ASD) and schizophrenia or psychotic spectrum). The substantial expansion of structural and functional connectivity research over the past 40 years has shown that both ASD and psychotic spectrum disorders have also been conceptualized as disorders of brain circuitry. Moreover, and particularly for ASD, this research was developed within a diagnostic time frame that itself underwent six revisions of the Diagnostic and Statistical Manual of Mental Disorders (DSM), from the third edition (DSM-III; 1980) to the fifth edition, text revision (DSM-5-TR; 2022), with the largest changes involving the elimination of the early language onset requirement and the consolidation of prior subtypes into a single autism spectrum disorder. The present review develops a mechanistic account of cerebral connectivity differences in autistic disorder as defined under DSM-III and DSM-IV, where diagnostic practice, and in particular the exclusion of clinically significant language delay from Asperger’s disorder, enriched cohorts for a subgroup in which cerebellar vermal lobule VI and VII abnormality, posterior callosal reduction, and atypical predictive processing were originally identified. The present account proposes, as a hypothesis rather than as an established finding, that deviation of vermal lobules VI and VII from typical development, in the direction of either hypoplasia or hyperplasia, both reported within the same DSM-III/IV cohort, and on evidence consistent with prenatal origin, initiates a developmental cascade that may shape postnatal cerebral connectivity through disinhibition of deep cerebellar nuclei and altered excitatory drive to thalamocortical circuits during sensitive periods. Cross-condition evidence indicates that vermal abnormality also occurs in conditions with distinct primary diagnoses (Joubert syndrome, fragile X, Rett syndrome, Williams syndrome, schizophrenia), producing the autistic-disorder cluster only when the upstream perturbation also affects cortical context-integration substrates at the relevant developmental window. These conditions are treated as further instances of a common neurodevelopmental class rather than as separate kinds of disorder, with autistic disorder as the focal case because it is where the vermal findings were first identified. Transdiagnostic connectivity evidence spanning autism and schizophrenia cohorts is examined to establish whether the cerebellar account generalizes across the SMI class or is specific to autistic disorder, and the regional and directional distribution of the cerebellar findings in each condition is treated as the discriminating variable. The framework proposes the cerebellum as a substrate for predictive internal models scaffolding auditory, social, and contextual learning, and is empirically testable through infant connectivity and event-related potential studies and through stratified re-analysis of multisite samples.

## 1. Introduction

### 1.1. Four Decades of Brain Circuitry Research Within a Shifting Diagnostic Frame

The diversity of social, language, behavioral, and cognitive symptoms and traits observed in individuals with neurodevelopmental disorders is now well recognized. Severe mental illness (SMI) is used throughout this review to denote the class of neuropsychiatric conditions that produce substantial and sustained impairment in functioning. The class encompasses disorders of adult onset, such as the psychotic spectrum disorders, together with those whose symptoms emerge in early childhood, such as autism. Over the past four decades, autism has increasingly been conceptualized as a neurodevelopmental disorder of brain circuitry, as have the genetic syndromes, attention-deficit/hyperactivity disorder, and the psychotic spectrum disorders, all of which are now widely understood to have neurodevelopmental origins even where the clinical presentation emerges in adolescence or adulthood [1,2,3]. The present review treats these conditions as members of a common neurodevelopmental class rather than as categorically separate kinds of disorder. Within that class, autism is the focal case, and specifically autistic disorder as defined under DSM-III and DSM-IV, because it is where the cerebellar vermal findings were first identified and where the diagnostic stratification that makes them interpretable was in force. The other conditions are examined not as peripheral comparisons but as further instances of the same developmental logic, each of which tests how specific the cerebellar account is to autism and how far it generalizes. During the same time frame, and arguably due to greater investigation involving autism, divergent patterns of neurocircuitry have also been found for each disorder. Methodological advances in structural magnetic resonance imaging, diffusion tensor imaging, resting-state functional magnetic resonance imaging, event-related potential recording, and large-scale multisite collaboration have substantially expanded what can be measured and inferred about neural network organization in autism [4,5,6]. The resulting empirical base now spans structural connectivity, functional connectivity, effective connectivity, and the dynamic and developmental trajectories of all three. This research, however, has been conducted in a context that warrants explicit acknowledgment before the connectivity findings can be appropriately interpreted: research cohorts have largely been assembled cross-sectionally, at successive points along a four-decade arc during which the diagnostic criteria for autism themselves underwent substantial revision.

#### 1.1.1. Severe Mental Illness: Scope of the Term and Rationale for the Grouping

Grouping autism with the psychotic spectrum disorders under a single heading requires justification, because the two are separated in clinical practice by roughly two decades of typical age at presentation. The justification is developmental rather than symptomatic. The neurodevelopmental account of schizophrenia holds that the pathogenic processes underlying psychosis are set in motion during fetal and early postnatal brain development, long before psychotic symptoms appear, and that the adolescent or early-adult onset of frank psychosis reflects the point at which a compromised system is loaded by normal maturational demands rather than the point at which the pathology begins [1,2,3]. On that account, adult onset is a fact about when a condition becomes clinically visible, not a fact about when it originates. If that is correct, then autism and schizophrenia differ in the nature and timing of an early developmental perturbation, and in the cortical context in which it occurs, rather than in whether the perturbation is developmental at all.

This grouping is what makes the cerebellar question tractable across conditions. If prenatal deviation of the cerebellar vermis were specific to autism, it would be a fact about autism. If it occurs across the SMI class but yields different phenotypes, then it is a fact about a shared developmental window, and the phenotypic divergence has to be explained by what else is happening in the cortex at the time. The second possibility is the one this review develops, and it is testable precisely because the conditions in the class have been studied with the same imaging methods against the same anatomical landmarks.

Two boundaries on the scope of the term should be stated at the outset. First, SMI is used here as an organizing category for a developmental argument and not as a diagnostic entity; no claim is made that the conditions grouped under it share a diagnosis, a treatment, or a prognosis. Second, the depth of coverage across the class is deliberately uneven. Autistic disorder as defined under DSM-III and DSM-IV is the focal case throughout, because that is where the cerebellar vermal findings were first identified and where the diagnostic stratification that makes them interpretable was in force. The psychotic spectrum disorders receive sustained treatment in a later section as the principal test of whether the account generalizes. The genetic syndromes and attention-deficit/hyperactivity disorder are treated more briefly, as cross-condition evidence bearing on the specificity of the cerebellar finding rather than as cases developed in their own right.

#### 1.1.2. The Diagnostic and Statistical Manual and Its Role in This Literature

The Diagnostic and Statistical Manual of Mental Disorders (DSM), published by the American Psychiatric Association and now in its fifth edition, text revision [7], provides the standardized diagnostic criteria by which research cohorts in this literature have been assembled and is therefore central to the argument that follows. The DSM specifies, for each disorder, the set of observable features that must be present for a diagnosis to be assigned, and researchers recruit participants by applying those criteria. Because the criteria are periodically revised as clinical understanding changes, the edition in force at the time a study was conducted is itself a determinant of who was enrolled in that study. Two investigations of the same nominal disorder, conducted twenty years apart, may therefore have studied materially different populations. The revisions summarized in Table 1 make this concrete for autism.

#### 1.1.3. A Note on Diagnostic Terminology

A terminological convention is followed throughout. Each study is described using the diagnostic label its own authors used, so that work reporting on autistic disorder is described in those terms and work reporting on autism spectrum disorder is described in its own. No attempt is made to retrofit contemporary terminology onto older cohorts, or older terminology onto contemporary ones. This convention has the advantage of being verifiable by any reader against the primary source, and it also renders visible the terminological drift that is itself part of the subject matter of this review.

Where the argument requires a claim about cohort composition rather than about nomenclature, that claim is made explicitly rather than carried implicitly by the label. Two such claims are made. Samples ascertained under DSM-III or DSM-III-R criteria are treated as having early language abnormality as a criterial feature, since gross deficits in language development were required for a diagnosis of infantile autism under DSM-III and impairment in communication was required under DSM-III-R. Samples diagnosed with autistic disorder rather than Asperger’s disorder under DSM-IV are treated as enriched for early language delay. The basis for the second claim is the boundary between the two diagnoses rather than the autistic disorder criteria themselves: DSM-IV Criterion B required delay or abnormal functioning before age three in at least one of three domains, of which language used in social communication was one, but the diagnosis of Asperger’s disorder required the absence of clinically significant general language delay. Cohorts assigned to autistic disorder rather than to Asperger’s disorder under DSM-IV were therefore selected against normal early language, whether or not each individual study reported language onset directly. Samples ascertained under DSM-5 or DSM-5-TR carry no such assumption, and that difference is itself among the things this review argues about.

#### 1.1.4. What DSM-5 Requires, and What It Does Not

The consequence of the DSM-5 revision for sample composition should be stated precisely, because it is easy to overstate. DSM-5 does require persistent deficits in social communication and social interaction across multiple contexts, encompassing deficits in social–emotional reciprocity, in nonverbal communicative behaviors used for social interaction, and in developing, maintaining, and understanding relationships. Pragmatic difficulty is therefore a criterion under DSM-5 and remains so under DSM-5-TR. What the 2013 revision removed was the requirement of delayed or deviant early language development. Language status was relocated to a specifier, recorded as with or without accompanying language impairment, and is no longer criterial for the diagnosis itself.

Two consequences follow for the literature reviewed here. The first concerns cohort composition. An autism spectrum disorder diagnosis under DSM-5 may be assigned to an individual whose early language development was entirely typical, which was not possible for infantile autism under DSM-III or autistic disorder under DSM-III-R, and which was constrained under DSM-IV by the requirement that Asperger’s disorder be diagnosed in the absence of clinically significant language delay. Contemporary cohorts therefore contain a smaller proportion of the language-delayed phenotype in which the cerebellar vermal findings were originally established, and that proportion has continued to shift as ascertainment has broadened. Analyses pooling across the full DSM-5 spectrum will dilute any effect specific to that subgroup, and the dilution will deepen over time. This is the case-mixing mechanism stated in terms of what the diagnostic criteria require rather than in terms of prevalence trends.

The second consequence is more useful, because it identifies a remedy available within existing data. Language impairment is recorded as a DSM-5 specifier rather than discarded, and early language onset items are collected as part of the Autism Diagnostic Interview, Revised in many multisite cohorts. The stratification this framework requires can therefore be performed retrospectively on data already gathered, without new recruitment. The resulting prediction is specific and falsifiable. Partitioning DSM-5 samples by early language status should recover the vermal lobule VI and VII effect within the language-impaired stratum, and should show its attenuation within the stratum without language impairment. Failure to recover the effect under stratification would count as evidence against the framework rather than as a further instance of the inconsistency the framework is intended to explain.

This prediction is not untested. D’Mello, Moore, Crocetti, Mostofsky, and Stoodley [8] partitioned a sample of autistic children by history of early language delay and reported that cerebellar gray matter differentiated the two groups, with the language-delayed subgroup showing the reduction. That study performs, within a contemporary sample, the stratification this review argues the field has largely failed to perform, and it recovers a cerebellar effect in an era whose pooled analyses report attenuation. It is therefore the strongest existing test of this specific stratification prediction, and its result is the one the present framework predicts. Two qualifications apply. The measure was regional gray matter volume rather than midsagittal vermal area, so the finding supports the stratification logic without directly confirming the vermal lobule VI and VII localization. A single study, however well aimed, does not settle the question. The appropriate conclusion is that the stratification approach was tried, that it worked, and that it warrants replication at the scale the large multisite datasets now permit.

Between the publication of DSM-III in 1980 and DSM-5-TR in 2022, the criteria for autism were revised six times (DSM-III in 1980 [9]; DSM-III-R in 1987 [10]; DSM-IV in 1994 [11]; DSM-IV-TR in 2000 [12]; DSM-5 in 2013 [13]; and DSM-5-TR in 2022 [7]). The largest changes involved the language and communication domain. The three editions differ from one another in ways that matter for the argument developed here, and it is worth stating them precisely. DSM-III [9] required gross deficits in language development as a criterion for infantile autism, with onset before 30 months. DSM-III-R [10] replaced that structure with a polythetic list requiring eight of sixteen items distributed across three domains, so that language impairment was highly probable in any given case but was not strictly required. DSM-IV [11] and DSM-IV-TR [12] after it required onset before three years of age in at least one of three areas: social interaction, language as used in social communication, or symbolic or imaginative play. Early language abnormality was therefore one qualifying route rather than a requirement of every DSM-IV autistic disorder diagnosis. What operated under DSM-IV was not the criterion itself but the categorical structure surrounding it. Asperger’s disorder, introduced in that edition, excluded clinically significant general language delay and cognitive delay by definition, and pervasive developmental disorder not otherwise specified absorbed subthreshold presentations, so cases presenting with early language delay were assigned to autistic disorder rather than to the adjacent categories. The consequence is probabilistic enrichment rather than strict selection. Historical autistic disorder cohorts contained a substantially higher proportion of individuals with early language abnormality than contemporary spectrum-defined cohorts do, without early language abnormality being a criterion in every case. Where this review uses the term stratification, that enrichment is what is meant, and the argument developed below does not require the stronger categorical claim. DSM-5, in 2013, consolidated these subtypes into a single autism spectrum disorder diagnosis and eliminated the language onset criterion entirely, replacing the prior triad with two domains (deficits in social communication and social interaction; restricted, repetitive patterns of behavior) and incorporating sensory atypicalities as a clinical feature. The restricted and repetitive behavior domain remained relatively stable across this transition, with the principal change in that domain being the explicit inclusion of sensory features in DSM-5. The social–communication domain underwent more substantial revision, with the consolidation away from specific language–topography criteria toward a more general framework emphasizing social–communication impairment across a spectrum of presentations.

Prevalence estimates spanning this period illustrate the population-level consequence of this diagnostic evolution. Identified autism prevalence in the United States rose from approximately 0.5 per 1000 children in 1980 under DSM-III, before national surveillance was established, to approximately 32 per 1000 in 2022 under DSM-5-TR as reported by Centers for Disease Control and Prevention surveillance, an approximately 64-fold difference, over a period in which diagnostic criteria, ascertainment practices, professional and public awareness, service eligibility, and surveillance methodology all changed [14,15,16,17,18,19]. These estimates derive from different case definitions and ascertainment systems, and do not constitute a single continuous surveillance series or a DSM-by-DSM natural experiment, and an association between revision and prevalence slope would not establish that criterion change produced the increase. The argument advanced here does not require that it did. What the record does establish is that comparisons between historical and contemporary cohorts are inherently vulnerable to differences in case composition. Whatever the relative contributions, the implication for connectivity research is direct: the population identified as having autism under DSM-5 criteria is substantially larger and biologically more heterogeneous than the population identified under DSM-III or DSM-IV.

The connectivity findings reviewed in this manuscript span this entire arc, and the diagnostic frame under which any given finding was originally reported is therefore a methodologically important feature of the literature. The foundational structural findings, including the report of vermal lobule VI and VII hypoplasia by Courchesne, Yeung-Courchesne, Press, Hesselink, and Jernigan [20], the bidirectional vermal pattern (approximately 86% hypoplastic, approximately 12% hyperplastic) reported by Courchesne, Saitoh, Yeung-Courchesne, Press, Lincoln, Haas, and Schreibman [21], the cerebellar hemisphere size correlations reported by Murakami, Courchesne, Press, Yeung-Courchesne, and Hesselink [22], the early cerebral and cerebellar overgrowth pattern reported by Courchesne et al. [23], and the posterior callosal reduction documented by Egaas, Courchesne, and Saitoh [24] were established in cohorts ascertained under DSM-III, DSM-III-R, and early DSM-IV criteria that required the language onset feature and thereby effectively stratified samples for what would now be classified as the autistic disorder subgroup with delayed language. Contemporary multisite cohorts, including the Autism Brain Imaging Data Exchange (ABIDE) I and II, were ascertained predominantly under DSM-IV-TR and DSM-5 criteria, without language onset stratification and across a broader phenotypic range. Replication of these foundational findings in contemporary multisite samples has been inconsistent rather than uniform, with several large-cohort analyses reporting attenuated or undetectable group-level effects [25,26,27] alongside other contemporary work that continues to identify cerebellar and connectivity differences in ASD samples [28,29,30,31]. The pattern of inconsistent replication has been variously interpreted, including in terms of methodological heterogeneity and true biological variability across the spectrum. A further interpretation, supported by independent evidence reviewed below, is that the foundational findings remain valid for the diagnostic cohort in which they were established but that pooled analyses across the broader DSM-5 spectrum dilute the signal through case-mixing. Two findings constitute the strongest pre-existing evidence consistent with the case-mixing interpretation. The Stanfield, McIntosh, Spencer, Philip, Gaur, and Lawrie [32] meta-analysis showed significant IQ moderation of the cerebellar effect in meta-regression analyses, with larger effects in the lower-IQ samples characteristic of older autistic disorder cohorts. Lincoln, Courchesne, Allen, Hanson, and Ene [33] demonstrated preserved vermal volume in seven Asperger syndrome case studies drawn from the same San Diego.

The developmental cascade and predictive-modeling framework developed below is offered as an account of the autistic disorder phenotype as defined under DSM-III and DSM-IV criteria, where the categorical structure of the diagnosis, and in particular the exclusion of early language delay from Asperger’s disorder, enriched samples for the subgroup in which the cerebellar and connectivity findings were originally identified. The framework is not offered as an account of the full DSM-5 autism spectrum, which on the present account is heterogeneous in ways that do not map cleanly onto any single mechanistic substrate. However, the framework does make specific and testable predictions about which DSM-5 individuals should show the cerebellar and connectivity signatures of interest: those with retrospective language onset delay (recoverable from Autism Diagnostic Interview, Revised early language items in many DSM-5 cohorts) and bidirectional vermal lobule VI and VII abnormality. The framework also explicitly accommodates cross-condition evidence reviewed in later sections indicating that vermal abnormality occurs in several conditions with distinct primary diagnoses (Joubert syndrome, Dandy–Walker malformation, fragile X syndrome, Rett syndrome, Williams syndrome, Prader–Willi syndrome, schizophrenia), producing the autistic disorder cluster of features only when the upstream perturbation also affects cortical context-integration substrates at the relevant developmental window. Vermal abnormality is therefore most accurately positioned as a developmental-window marker rather than as a sufficient cause of the cluster of features defining autistic disorder.

A note on method and on vocabulary is in order before the evidence is presented. This is a mechanistic narrative review rather than a systematic review, and no formal search protocol or inclusion algorithm was applied. Studies were selected to represent foundational anatomical findings, contemporary multisite replication attempts, developmental and infant evidence, mechanistic animal models, predictive-processing theory, and transdiagnostic comparison, with preference given to work that bears on the specific claims advanced here rather than to comprehensive coverage of the cerebellar literature. Where the review describes evidence as convergent, the term refers to agreement across methods and laboratories, and where the underlying reports derive from overlapping cohorts or a common methodological lineage, this is noted. Three levels of claim are distinguished throughout and should not be read as interchangeable. The fact that the cerebellum can influence developing cortical systems is anatomically established. That early cerebellar dysfunction could alter cortical maturation is mechanistically plausible and supported by animal perturbation work. That vermal deviation is upstream of the cortical connectivity phenotype in autistic disorder remains a hypothesis, and the language used for it is hypothesis language throughout.

The sections that follow are organized to reflect this framing. The review first situates the present account within the broader shift in autism research from localization to connectivity, summarizes the three principal forms of brain connectivity that have been measured, and reviews the diagnostic considerations that bear on cross-cohort comparison. It then introduces the severe mental illness framing and the role of the DSM in shaping the cohorts under discussion. The review next describes the structural and functional connectivity findings in autistic disorder, the cerebellar and vermal abnormalities that distinguish the autistic disorder phenotype, the evolutionary origins of the neocerebellar regions implicated, and the cross-condition pattern of vermal abnormality. Cerebellar evidence from the psychotic spectrum disorders is then examined, as the principal test of how far the account generalizes across the severe mental illness class. A briefer comparison with obsessive–compulsive disorder follows, included for the circuit it implicates rather than as a survey of that literature. The proposed developmental cascade and its molecular and synaptic substrates are then developed, formalizing the cerebellar contribution to predictive modeling, and close with testable predictions and recommended priorities for future research. Chief among the priorities this framework generates is the stratified re-analysis of large multisite samples, partitioned by early language onset and by direction of vermal deviation rather than pooled across the full DSM-5 spectrum. Throughout, references to “autism” and to “autistic disorder” reflect the diagnostic frame of the cited source: contemporary references using “autism spectrum disorder” or “ASD” reflect DSM-IV-TR or DSM-5 ascertainment, while references using “autistic disorder,” “infantile autism,” or “autism” in older sources predominantly reflect DSM-III or DSM-IV ascertainment of the language-delayed subgroup. This distinction is made explicit where it bears on the interpretation of a finding.

### 1.2. From Localization to Connectivity in Autism Research

The human brain comprises approximately 86 billion neurons [34,35] interconnected through trillions of synapses [36], forming complex networks that appear to support most aspects of human cognition, emotion, and behavior. Historically, neuroscience often adopted a localizationist perspective, attempting to map specific functions to discrete brain regions [37]. The discovery by Biswal et al. [5] that the human sensorimotor system displayed spontaneous synchronized activity during rest, termed resting-state connectivity, contributed to a shift toward viewing the brain as an interconnected network rather than a collection of isolated modules [38,39].

This network perspective has proven particularly informative for understanding neurodevelopmental conditions such as autism. Early descriptions by Kanner [40] emphasized social impairments in autism, derived from the Greek term auto, meaning self, reflecting how affected individuals appeared to be lost in their own narrow worlds. Modern neuroimaging suggests that these behavioral characteristics may correspond to differences in how brain networks are organized and function [6,41,42,43,44,45], supporting a view of autism as a distributed neural systems condition rather than focal brain pathology [46,47,48,49].

Among the candidate mechanisms for these distributed network differences, the cerebellum has emerged as a structure of particular interest. Once viewed primarily as a motor coordinator, the cerebellum is now recognized as a participant in distributed cognitive, affective, and social networks [50,51,52,53,54,55]. It is also a region consistently implicated in the neuropathology of autism [20,21,56,57,58,59,60,61,62]. Two foundational observations frame the present account. First, structural studies of autistic disorder have repeatedly identified hypoplasia of cerebellar vermal lobules VI and VII, with the reduction more pronounced and developmentally early in autistic disorder than in any other neurodevelopmental condition examined to date [20,21,22,32,63,64,65]. Second, neocerebellar regions including lobules VI and VII and their hemispheric extensions (Crus I and Crus II) are phylogenetically recent expansions that show selectively elevated growth in great apes and humans [66,67,68], supporting their candidate role in functions that distinguish autistic from typical cognitive and social development.

This review develops a mechanistic account in which prenatal cerebellar vermal hypoplasia initiates a developmental cascade that shapes postnatal cerebral connectivity through several specific routes. The general proposition that early cerebellar disruption shapes subsequent cortical development was originally articulated by Courchesne and colleagues on the basis of the structural findings of the late 1980s and early 1990s [69,70,71,72]. Multiple independent laboratories have since developed and extended it, in directions ranging from animal-model genetics through human neuroimaging to computational theory [56,58,59,60,62,73,74,75]. In the present account, disrupted Purkinje cell development reduces inhibitory drive to the deep cerebellar nuclei, increasing excitatory output through cerebello-thalamo-cortical pathways during sensitive periods of postnatal cortical maturation. This altered excitatory drive contributes to three outcomes. The first is the characteristic ASD pattern of local cortical overconnectivity with reduced long-range integration [41,46,47,49,76,77,78]. The second is the posterior corpus callosum reduction documented in autistic disorder by independent laboratories [24,79,80,81,82]. The third is compromised generation of the forward predictive models that, in typical development, scaffold rapid learning across auditory, social, and contextual domains [51,53,54,55,83]. The framework draws on (a) classical neuroanatomical findings from autistic disorder across multiple laboratories, (b) recent evidence from neonatal and infant functional connectivity studies, (c) comparative primate evidence on cortical and cerebellar architecture, (d) experimental work in genetic and circuit-level mouse models, and (e) the predictive-coding literature articulating the cerebellum’s role in generating forward internal models.

**Figure 1:**
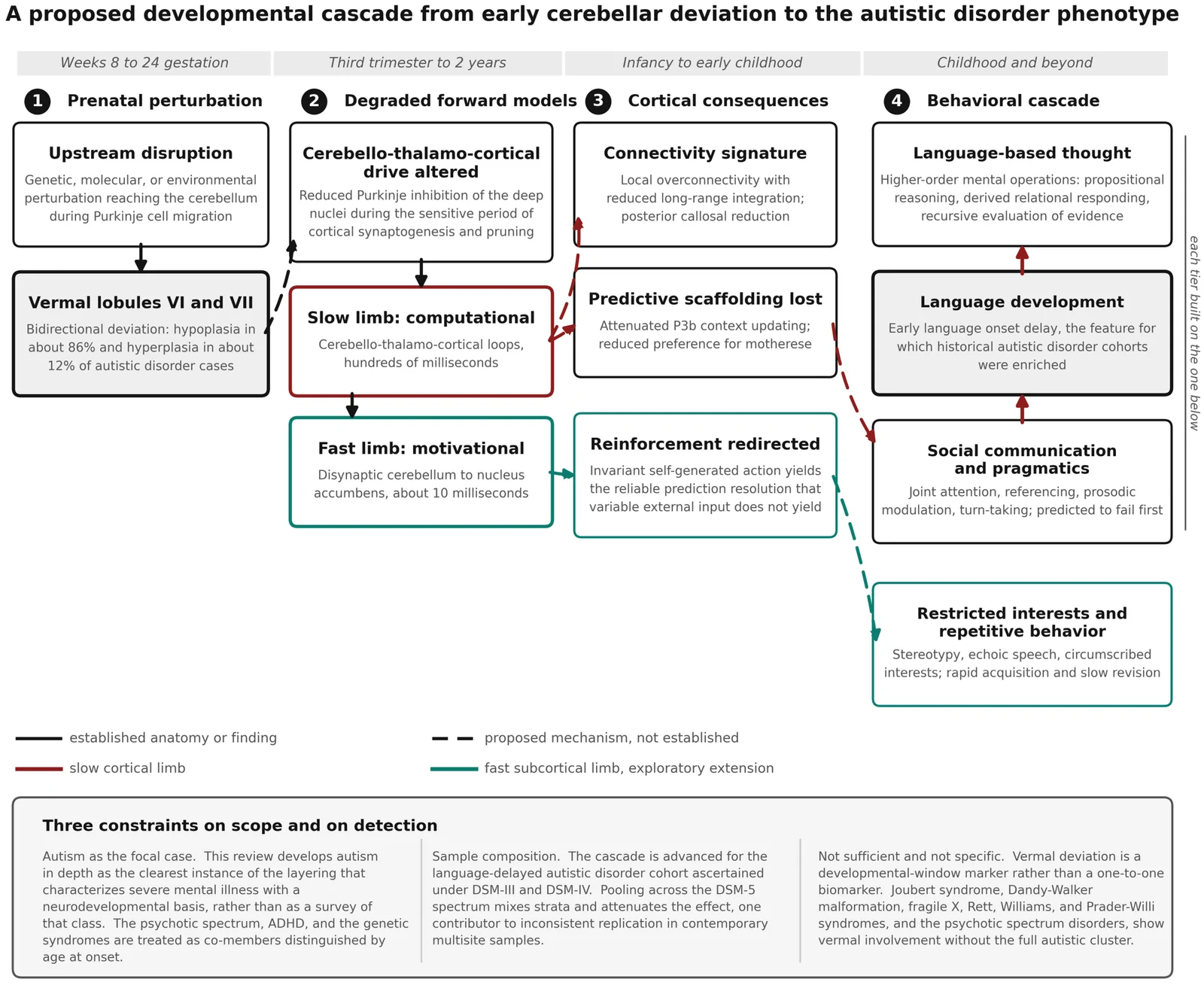
Overview of the proposed developmental cascade and predictive-modeling framework developed in this review. Line style encodes evidentiary status throughout: solid arrows mark established anatomical connections or replicated findings, and dashed arrows mark proposed mechanisms that the review advances as hypotheses rather than as demonstrated relations. On the account proposed here, an upstream genetic, molecular, or environmental perturbation reaching the cerebellum during Purkinje cell migration produces bidirectional deviation of vermal lobules VI and VII, reported as hypoplasia in approximately 86 percent and hyperplasia in approximately 12 percent of an autistic disorder sample. Reduced Purkinje inhibition of the deep nuclei alters excitatory drive through cerebello-thalamo-cortical pathways during the sensitive period in which cortical synaptogenesis peaks and activity-dependent pruning remains active. Cerebellar output reaches downstream systems by two routes that differ by roughly two orders of magnitude in latency. The slow cortical limb, shown in dark red, operates through cerebello-thalamo-cortical loops on a timescale of hundreds of milliseconds and yields the cortical connectivity signature of local overconnectivity with reduced long-range integration and posterior callosal reduction, together with the loss of predictive scaffolding indexed electrophysiologically by attenuated P3b context updating and behaviorally by reduced preference for infant-directed speech. The fast subcortical limb, shown in teal, is the disynaptic cerebellum-to-nucleus-accumbens projection with an estimated conduction time of approximately 10 milliseconds in the mouse, and it redirects reinforcement toward self-generated invariant action. The behavioral cascade at right is ordered by developmental precedence. Social communication and pragmatics fail first, because joint attention, referencing, prosodic modulation, and turn-taking are the operations through which the contingency structure of social exchange is first sampled. Language development follows, with early language onset delay serving as the DSM-III and DSM-IV criterion that defined the cohorts in which the vermal findings were established. Language-based thought and the higher-order mental operations that language makes possible sit above language and inherit the impoverished input of the tiers below. Restricted interests and repetitive behavior, including stereotypy and echoic speech, are shown as the separate consequence of the fast limb rather than as a further tier of the language cascade. The lower band states three constraints. Autism is developed here as the focal case within severe mental illness rather than as a survey of that class; the cascade is advanced for the language-delayed autistic disorder cohort rather than for the full DSM-5 spectrum; and vermal deviation functions as a developmental-window marker rather than as a one-to-one biomarker. The figure is a schematic of the proposed relations rather than an exhaustive circuit diagram, and the ordering of the behavioral tiers is a prediction of the model rather than an established developmental sequence.

Two further considerations cut across this account. First, the diagnostic-frame issue introduced in the preceding section bears throughout. The DSM-IV-era distinction between autistic disorder and Asperger syndrome is maintained wherever the cited literature supports it, particularly at the level of cerebellar and callosal anatomy [21,33]. The contrast with developmental receptive language disorder, in which lower-level auditory abnormalities co-occur with intact higher-order predictive processing, is treated as a useful constraint on the framework [84,85,86]. Second, methodological sources of variability across the connectivity literature, including analytic approach, head motion, sample composition, and multisite acquisition effects, contribute substantially to apparent inconsistencies and warrant explicit attention. Drawing on the foundational work of Courchesne, Lincoln, and colleagues alongside contemporary connectivity findings and theoretical models, this review develops an account that is developmentally grounded, mechanistically specific, and empirically testable. Figure 1 provides an overview of the account in a single view, from the prenatal perturbation through the two limbs of cerebellar output to the ascending tiers of the behavioral cascade, and may be used as a map for the sections that follow.

### 1.3. Types of Brain Connectivity

Brain connectivity research examines three fundamental types of neural relationships, each providing complementary information about brain organization.

Structural connectivity refers to the physical white matter pathways enabling information transfer between brain regions [87], most commonly assessed using diffusion tensor imaging (DTI) and related diffusion-weighted methods that track water diffusion along fiber bundles [88,89]. Standard DTI metrics such as fractional anisotropy index white matter microstructure [90,91], with applications across developmental, surgical, and clinical contexts [4,92]. Important limitations include the inability of the tensor model to resolve crossing fibers within a voxel, the composite nature of fractional anisotropy as an index of axonal density, myelination, and free-water contamination, and tractography susceptibility to false positives and false negatives in long-range pathway reconstruction [93,94,95]. More recent diffusion models, including high angular resolution diffusion imaging, fiber orientation distribution tractography, and neurite orientation dispersion and density imaging, address some of these limitations at the cost of longer acquisitions and more complex pipelines [96,97].

Functional connectivity measures statistical dependencies between spatially separated regions’ activity patterns [37], typically through temporal correlations in BOLD signal fluctuations during resting-state fMRI [5,38], yielding reproducible large-scale networks including the default mode, frontoparietal, salience, dorsal attention, and somatomotor systems [39,98]. Strengths include task-independence (feasible in young children and clinical populations), whole-brain coverage at 2 to 3 mm resolution, and reproducible group-level topographies across sites [50,99]. Limitations include the hemodynamic proxy nature of BOLD with limited temporal resolution [100], sensitivity to head motion, physiological noise, and analytic choices [101], and the inability of correlations alone to establish causal influence [37]. Individual-level test–retest reliability is modest in standard acquisitions and substantially improved by scans of 25 min or longer [102,103].

**Figure 2:**
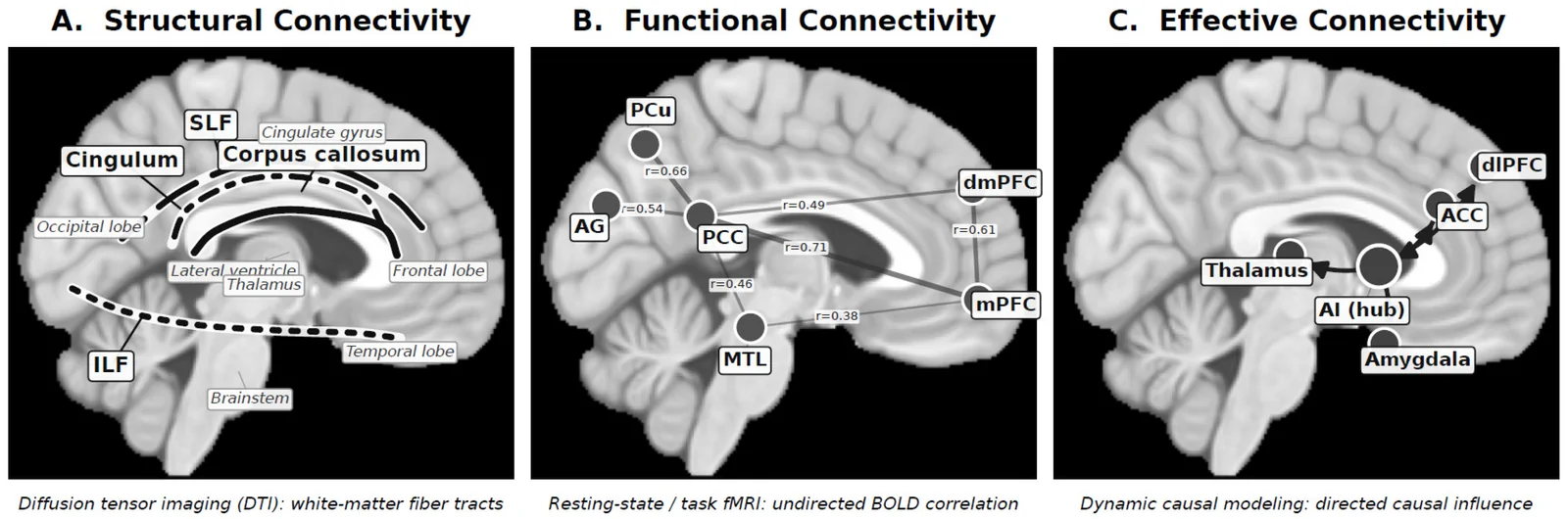
Schematic representation of the three principal forms of brain connectivity, illustrated on a sagittal slice of the MNI152 standard anatomical template (anterior is to the right). Panel (**A**). Structural connectivity, typically measured with diffusion tensor imaging (DTI), characterizes white matter fiber tracts that physically link brain regions. Four major tracts are identified by labeled arrows pointing to their location on the sagittal slice: the corpus callosum (body visible as the white C-shaped ribbon arching over the lateral ventricles), the largest commissural tract connecting homologous regions of the two cerebral hemispheres; the superior longitudinal fasciculus (SLF), a major association tract connecting frontal with parietal, temporal, and occipital cortex; the cingulum bundle, which runs above the corpus callosum within the cingulate gyrus and supports limbic and default mode network communication; and the inferior longitudinal fasciculus (ILF), a ventral association tract connecting occipital and anterior temporal cortex. The SLF and ILF run primarily in lateral white matter and are projected schematically onto the medial sagittal plane for illustration. Panel (**B**). Functional connectivity, typically measured with resting-state or task-based fMRI, is defined by temporal correlation of blood-oxygen-level-dependent (BOLD) signal between regions and is undirected. The default mode network is shown for illustration, with six representative nodes: medial prefrontal cortex (mPFC), dorsomedial prefrontal cortex (dmPFC), posterior cingulate cortex (PCC), precuneus (PCu), angular gyrus (AG), and medial temporal lobe (MTL). The angular gyrus, a lateral parietal structure, is shown at its approximate projection onto the medial sagittal plane. Edges depict pairwise Pearson correlations between regional BOLD time series, with the width and saturation of each edge proportional to the magnitude of the correlation coefficient r; illustrative r values shown range from approximately 0.38 to 0.71, consistent with the range typically observed for intra-default-mode-network connectivity in young adult samples [109,110]. Panel (**C**). Effective connectivity, often estimated with dynamic causal modeling or Granger-causality-based approaches, captures the directional influence one region exerts over another. The salience network is illustrated, with directed arrows depicting causal influences among five representative nodes: anterior insula (AI), anterior cingulate cortex (ACC), dorsolateral prefrontal cortex (dlPFC), thalamus, and amygdala. The anterior insula is shown as a hub region that exerts driving influence on multiple downstream targets and receives reciprocal feedback from the anterior cingulate cortex, consistent with proposals that the salience network coordinates switching between default mode and central executive systems. Lateral structures (AI and dlPFC) are positioned at their approximate projection onto the medial sagittal plane for illustration, and the dlPFC is shown as an interacting executive-network region rather than a canonical salience-network node. Together, the three panels illustrate that structural, functional, and effective connectivity offer complementary perspectives on brain network organization: anatomical pathways, undirected temporal coupling, and directional causal influence, respectively. Abbreviations: AG, angular gyrus; AI, anterior insula; ACC, anterior cingulate cortex; BOLD, blood-oxygen-level-dependent; dlPFC, dorsolateral prefrontal cortex; dmPFC, dorsomedial prefrontal cortex; DTI, diffusion tensor imaging; fMRI, functional magnetic resonance imaging; ILF, inferior longitudinal fasciculus; mPFC, medial prefrontal cortex; MTL, medial temporal lobe; PCC, posterior cingulate cortex; PCu, precuneus; SLF, superior longitudinal fasciculus.

Effective connectivity examines causal influences one neural system exerts over another, revealing directional information flow rather than only co-activation [104,105]. Common approaches include dynamic causal modeling [104], Granger causality applied to fMRI or electrophysiology [106], and regression dynamic causal modeling for whole-brain effective connectomes [107]. The principal strength is that, unlike correlation-based methods, effective connectivity can, in principle, distinguish driver from receiver. Limitations include model dependence, hemodynamic sensitivity, historical computational restriction to a small number of regions, and uncertainty about whether inferred causal relationships reflect neural causation or modeling assumptions [37,108]. These three forms of connectivity, and the level at which each is measured, are summarized in Figure 2.

## 2. Atypical Brain Connectivity: Autism Spectrum Disorder

### 2.1. Diagnostic Considerations: Pre-DSM-5 Versus DSM-5 Nomenclature

Comparison of connectivity findings across studies requires attention to the diagnostic frameworks under which participants were recruited. Prior to the publication of DSM-5 in 2013, the DSM-IV and DSM-IV-TR [11,12] maintained distinct diagnostic categories within the broader umbrella of pervasive developmental disorders, including autistic disorder, Asperger’s disorder, childhood disintegrative disorder, and pervasive developmental disorder not otherwise specified (PDD-NOS). The DSM-IV criteria for autistic disorder were notably specific. They required impairment in social interaction, impairment in communication, and restricted, repetitive, and stereotyped patterns of behavior, with delays or abnormal functioning in at least one of the following areas, with onset prior to 3 years of age: social interaction, language as used in social communication, or symbolic or imaginative play [12]. The communication criterion was particularly distinctive, requiring at least one of several qualifying manifestations: delayed or absent spoken language not compensated for by alternative modes of communication, marked impairment in initiating or sustaining conversation, or stereotyped, repetitive, or idiosyncratic language. Early language delay was therefore one qualifying route into the diagnosis rather than a requirement in every case.

Older neuroimaging investigations of autism, including the foundational cerebellar work by Courchesne and colleagues [20,21] discussed below, selected participants under these earlier criteria. The consequence is that such samples were enriched for individuals with delayed and atypical early language. The DSM-5 [13] consolidated the prior categories into a single autism spectrum disorder diagnosis and removed delayed language onset as a defining feature, replacing the DSM-IV triad with two symptom domains (deficits in social communication and social interaction; restricted, repetitive patterns of behavior) and incorporating sensory atypicalities as a clinical feature. The DSM-5 criteria therefore capture a broader and more heterogeneous group, including many individuals without language delay who would previously have been diagnosed with Asperger’s disorder or PDD-NOS rather than autistic disorder.

The implication for connectivity research is that comparisons between older and newer studies are not straightforward. Findings reported in samples meeting DSM-III or DSM-IV criteria for autistic disorder may not generalize uniformly to all individuals carrying a current DSM-5 ASD diagnosis, particularly with respect to neural systems supporting language development. Conversely, recent connectivity findings derived from more inclusive DSM-5 samples may dilute or obscure effects observed in earlier, more language-impaired samples. Where possible, attention to the diagnostic framework under which a study’s sample was characterized aids in interpreting cross-study consistencies and discrepancies in connectivity findings.

### 2.2. Structural Connectivity Alterations in ASD

#### 2.2.1. Developmental Divergence in White Matter

White matter development in autism may follow an atypical developmental trajectory. Structural magnetic resonance imaging studies have reported early enlargement of cerebral white matter, followed by slower or arrested growth later in childhood in some samples [23,111,112,113,114,115,116,117]. In 2- to 3-year-old children with ASD, fractional anisotropy has been reported as increased in frontal lobes and corpus callosum, suggesting early white matter overgrowth or altered organization. By age 5, this pattern appears to reverse: fractional anisotropy becomes reduced in frontal lobe tracts compared to typically developing children, with no clear differences observed in tracts connecting frontal and posterior regions in this age range. By ages 10 to 18, individuals with ASD have shown evidence of reduced fractional anisotropy in frontal-posterior connections, indicating possibly compromised long-range structural connectivity.

The qualification matters. The evidence does not support a single trajectory of early overgrowth followed by slowed growth through which every autistic child passes. Heterogeneity is substantial across age, sex, brain region, and sample. Mak-Fan, Morris, Vidal, Anagnostou, Roberts, and Taylor [118], examining developmental change in diffusion measures across white matter tracts in children with autism spectrum disorder, found age-by-group differences in frontal, long-distance, interhemispheric, and posterior tracts, with the pattern varying by region rather than shifting uniformly. That regional variation is what the present framework would expect if the disturbance operates through altered cerebello-thalamo-cortical drive during a period when different tracts are maturing on different schedules, since the tracts maturing latest and depending most on distributed integration should be the most affected. It also means that the trajectory claim should be stated at the level of altered regulation of white matter growth rather than as a uniform overgrowth-then-arrest sequence.

This developmental trajectory suggests that autism may involve early brain overgrowth of white matter followed by reduction in adolescence and adulthood, with DTI revealing alterations in white matter pathways. Investigations by Courchesne and colleagues reported that abnormal brain overgrowth in autism during the first years of life appears related to enlargement of cerebral, cerebellar, and limbic structures, with increases in both gray and white matter volume, and pronounced increases in cerebral (approximately 18%) and cerebellar (approximately 39%) white matter [23]. The early brain overgrowth finding has been replicated by independent laboratories using different cohorts and methods: Hazlett et al. [113] reported increased cerebral cortical volume in toddlers with autism, Hazlett et al. [114] extended the finding to a larger longitudinal sample, Hazlett et al. [115] documented hyperexpansion of cortical surface area between 6 and 12 months of age in high-familial-risk infants who subsequently developed autism, Schumann et al. [116] characterized atypical growth trajectories of frontal and temporal lobes in children with autism, Shen et al. [117] reported extra-axial cerebrospinal fluid accumulation in autism toddlers, and Nordahl et al. [119] confirmed early brain enlargement in a prospective sample. Diffusion tensor imaging in children with ASD has also shown white matter compromise of callosal and subcortical fiber tracts [120,121], and Lewis et al. [122] and Frazier et al. [123] reported altered callosal fiber length, reduced interhemispheric connectivity, and meta-analytic confirmation of corpus callosum reduction across imaging studies, consistent with the brain overgrowth and underconnectivity hypothesis. Together, these findings, drawn from multiple independent laboratories, support the view that autistic and neurotypical brains differ in their developmental organization.

#### 2.2.2. Regional Patterns and Behavioral Correlations

Multimodal studies integrating DTI, fMRI, and structural MRI suggest that distinguishing connectivity features in ASD may appear in temporal, parietal, and occipital lobes from DTI, prefrontal and parietal lobes from fMRI, and temporal lobes from structural MRI. In some samples, these distinguishing connectivity features have been related to abnormal social interaction behaviors in autism. The combination of all three modalities reportedly achieved 82.69% accuracy in differentiating individuals with ASD from typically developing controls, exceeding any single modality or two-modality combination in that sample [124,125]. More recent graph-based and multi-template machine learning approaches have continued to refine these classification performances; for example, multi-graph cross-attention frameworks [126], spatio-temporal weighted multi-hypergraph networks [127], outlier-screening multi-view frameworks [128], and knowledge-guided large-language-model-augmented graph networks [129] have each reported improvements in ASD classification on the ABIDE dataset relative to earlier methods. Akshoomoff et al. [130] reported that a discriminant function analysis of MRI brain measures correctly classified 95.8% of ASD cases and 92.3% of control cases in their sample, with this set of variables also showing some predictive value for functional outcomes in children with autism.

#### 2.2.3. Cerebellar Network Alterations

The cerebellum has emerged as a region of particular interest in autism. Pappaianni et al. [131] used Source-Based Morphometry, a multivariate method based on Independent Component Analysis, to examine gray matter networks in 43 male children with ASD and 46 male controls aged 6 to 12 years drawn from the Autism Brain Imaging Data Exchange (ABIDE) database. Their analysis identified an independent component (IC2) that differed significantly between groups (*p* = 0.049), encompassing several cerebellar regions including the inferior semi-lunar lobule, tuber, pyramis, declive, uvula, and cerebellar tonsil, along with a small portion of the right fusiform gyrus. A second cerebellar network involving the inferior semi-lunar lobule and cerebellar tonsil bilaterally showed a similar but weaker trend (*p* = 0.079). A fronto-temporal network involving the insula, superior temporal gyrus, inferior frontal gyrus, and fusiform gyrus correlated positively with stereotyped behavior scores on the Autism Diagnostic Observation Schedule (ADOS; r = 0.38, *p* = 0.01).

These findings extended earlier work in older individuals [132] and converge with diffusion imaging investigations. Khan et al. [29] examined cerebro-cerebellar resting-state functional connectivity in children and adolescents with ASD, reporting altered connectivity patterns between cerebellar regions and supratentorial cortex in their sample. Lincoln, Lai, and Jones [133] had earlier reported dissociation of shifting attention and joint attention in Williams syndrome, suggesting that cerebellar involvement may relate to social deficits across neurodevelopmental conditions. Larger-scale multisite work has further reinforced these findings: Oldehinkel et al. [31], drawing on the EU-AIMS Longitudinal European Autism Project (*n* = 265 ASD; *n* = 218 typically developing), reported decreased connectivity between the visual association network and somatosensory, medial, and lateral motor networks alongside increased connectivity between the cerebellum and these sensory and motor networks in ASD, with autism trait severity dimensionally associated with within-network connectivity of salience, medial motor, and orbitofrontal networks. The cumulative evidence supports a view of the cerebellum as engaged in a range of cognitive functions, including language, auditory processing, visuospatial perception, verbal memory, sequencing, and executive functions, making cerebellar alterations potentially relevant to the broad phenotype observed in ASD.

Subsequent investigations have refined the picture by identifying specific cerebellar subregions, networks, and developmental trajectories implicated in autism. Hanaie et al. [28] examined resting-state functional connectivity between cerebellar subregions (hemispheric, vermal, and dentate nucleus) and cerebral cortices in children and adolescents with ASD across two independent samples, with findings of aberrant connectivity converging on the posterior cerebellar hemisphere, the right dentate nucleus, and the posterior inferior vermis, and associating with motor, executive, and socio-communicative functions. Bednarz and Kana [134] extended these observations to the level of canonical intrinsic connectivity networks, reporting stronger functional connectivity between the ventral default mode network and left cerebellar lobules I–IV in children with ASD aged 7 to 12 years, indicating that cerebellar contributions to autism connectivity are not confined to neocerebellar regions but extend across the cerebellar mantle. Olivito et al. [30], in adults with ASD, identified reduced gray matter volume in right Crus II, a region implicated in social cognition, and found that this volumetric reduction tracked the degree of autistic traits as measured by the Autism-Spectrum Quotient, with corresponding alterations in functional connectivity between reduced Crus II and contralateral frontal and temporal cortex. Anteraper, Guell, Taylor et al. [135] and Arnold Anteraper, Guell, D’Mello et al. [136] reported abnormal resting-state functional connectivity of the dentate nuclei in high-functioning ASD, with seed-based analyses identifying two cerebellar clusters (Crus I/II and lobule IX) showing significant underconnectivity with cerebral cortical regions involved in social, emotional, and language processing, and with dentato-cerebral connectivity correlating with ASD symptom severity. These findings are notable in light of the cascade model developed below: the dentate nucleus is the principal output of the cerebellar hemispheres, and its disruption is precisely what the cascade framework predicts as a consequence of upstream Purkinje cell dysfunction.

Cortical–cerebellar circuits also show altered developmental trajectories in autism. Lepping et al. [137], examining visuomotor functional connectivity across an age range of 9 to 35 years, found greater age-associated increases in functional connectivity between cerebellum and visual, motor, and prefrontal cortical areas in ASD relative to controls, consistent with delayed maturation of cerebellar-cortical sensorimotor and nonsensorimotor networks. Unruh et al. [138] reported reduced functional connectivity between left anterior intraparietal lobule and right Crus I during visuomotor performance in ASD at high force levels, with this parietal-cerebellar reduction associated with more severe ASD symptoms; the authors interpreted these findings as reflecting deficits in integration of multimodal sensory feedback and reduced reliance on error-monitoring processes, a finding directly relevant to the predictive-modeling framework developed in a later section. Persichetti et al. [139], using a robust functional parcellation routine optimized for high temporal signal-to-noise ratio in 70 high-functioning ASD participants and 70 tightly matched controls, identified atypical functional networks in three convergent respects: whole-brain connectivity patterns were less stable across voxels within multiple functional networks, the cerebellum, subcortex, and hippocampus showed weaker differentiation of functional subnetworks, and these regions were atypically integrated with the neocortex. The convergence of these findings across imaging methods, age ranges, and analytic approaches supports cerebellar-cortical connectivity disruption as a robust feature of autism rather than an artifact of any single method.

The role of the cerebellum in autism has been the subject of multiple recent reviews. Sydnor and Aldinger [61] provided a comprehensive integration of structural, functional, and genetic contributions of the cerebellum to autism, emphasizing its protracted developmental timeline and the high density of neurons it contains, organized through complex connections with cortical and subcortical regions that subserve sensorimotor, executive, and limbic functions. The reviews converge on the conclusion that cerebellar dysfunction is a candidate unifying theory of autism, though one whose specific mechanistic links to behavioral phenotypes remain under active investigation. While the emphasis of this review focuses on autism, analogous findings can be found in other neurodevelopmental and genetic disorders, and in the psychotic spectrum disorders such as schizophrenia and bipolar disorder, which are themselves increasingly understood in neurodevelopmental terms. Cerebellar abnormalities have been reported in schizophrenia to include reduced cerebellar volume in both gray and white matter thickness involving lobules IV, V, VII, and crus I, II, and VIII; and in bipolar disorder to involve the right cerebellar hemisphere, including lobules I to IV, V, and crus I and II [140]. The developmental cascade and predictive-modeling frameworks elaborated in the following subsections offer one such mechanistic specification.

#### 2.2.4. Evolutionary Origins of the Neocerebellum

The cerebellar regions implicated in autism connectivity findings have specific evolutionary origins that distinguish them from older cerebellar divisions. The cerebellum can be partitioned phylogenetically into three divisions. The archicerebellum (lobule X, the flocculonodular lobe) is present in some form in all jawed vertebrates and is concerned with vestibular function. The paleocerebellum or spinocerebellum (anterior lobe, lobules I–V) is present across vertebrates and is concerned with sensorimotor integration. The neocerebellum or cerebrocerebellum (posterior lobe, lobules VI–VII and their hemispheric extensions) emerged with the development of substantial cortico-pontine connections and expanded markedly during mammalian and primate evolution [141,142,143]. This tripartite partition does not apply uniformly across the vermis, and the distinction matters for the lobule VI–VII findings reviewed below. The anterior vermis (lobules I through V, rostral to the fissura prima) belongs to the older, earlier-developing spinocerebellum. The posterior superior vermis (lobules VI and VII, between the fissura prima and the fissura prepyramidalis) is by contrast the midline component of the pontocerebellar neocerebellum. It receives corticopontine input, develops later [144], and aligns functionally with the cognitive rather than the sensorimotor cerebellum [145]. The posterior superior vermis, referred to throughout this review as the neocerebellar vermis, is therefore developmentally distinct both from the anterior vermis and from the adjacent sensorimotor lobule VIII, and it is the region in which Courchesne, Yeung-Courchesne, Press, Hesselink, and Jernigan [20] first identified vermal hypoplasia in autism.

Lobules VI and VII, together with their lateral hemispheric extensions (the ansiform lobule comprising Crus I and Crus II), are evolutionarily the youngest cerebellar regions. They are small or rudimentary in non-mammalian vertebrates and remain modest in size across most non-primate mammals. In primates, however, these regions show dramatic expansion. Comparative neuroimaging studies have demonstrated that the cerebellum, and particularly the hemispheric extensions of lobule VII (Crus I and Crus II), expanded substantially in the lineage leading to great apes and humans [67]. Rilling and Insel [68] reported that humans possess a relatively larger cerebellum than monkeys, with this difference particularly pronounced for the lateral cerebellar hemispheres. Barton and Venditti [66] extended this analysis using phylogenetic comparative methods, reporting that the cerebellum underwent rapid evolutionary expansion in great apes and especially humans, with rates of cerebellar change exceeding those of the neocortex in late hominoid evolution.

Importantly, this expansion appears to have been selective rather than uniform. Balsters et al. [146] demonstrated that the cerebellar lobules connecting with the prefrontal cortex, primarily Crus I and Crus II as the hemispheric extensions of vermal lobule VII, expanded disproportionately more than lobules connecting with motor cortex when comparing capuchin monkeys, chimpanzees, and humans. Weaver [147] provided additional support from analysis of fossil hominin endocasts, suggesting reciprocal evolution of cerebellum and neocortex during human evolution, with cerebellar expansion accelerating in late Pleistocene Homo. These findings position vermal lobules VI–VII and their hemispheric extensions as a phylogenetically recent neural substrate that became progressively elaborated in primate and human evolution, supporting cognitive and affective functions in addition to motor coordination. The evolutionary recency and primate-specific expansion of these regions make the consistent reporting of vermal VI–VII abnormalities in autism a particularly notable observation: disruption to a phylogenetically young, cognitively implicated region may produce more functionally consequential alterations than disruption to phylogenetically older regions whose roles in basic sensorimotor function are subserved by more conserved circuits.

#### 2.2.5. Vermal Lobules VI and VII: A Distinguishing Feature of Autism

**Figure 3:**
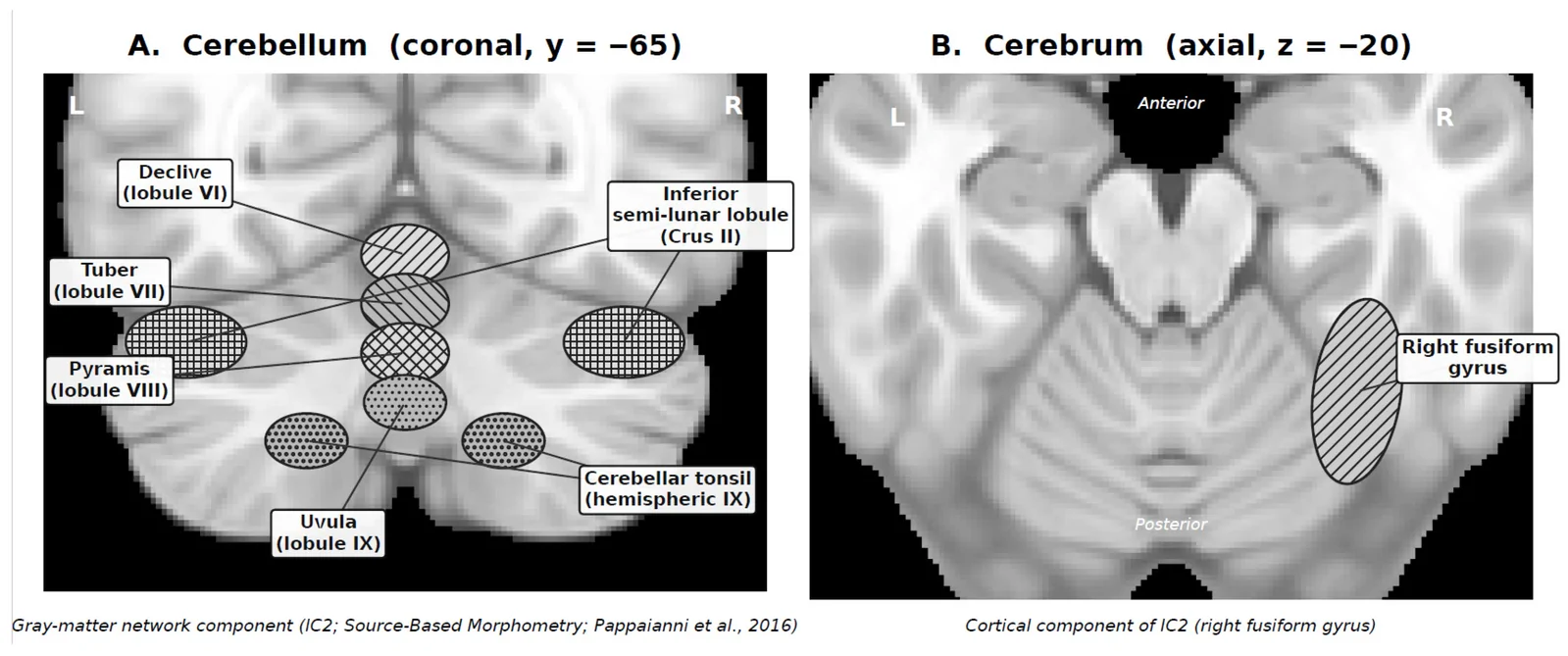
Cerebellar regions identified as showing altered gray matter network organization in children with autism spectrum disorder compared to typically developing controls (adapted from Pappaianni et al., 2016 [131]), shown on the MNI152 standard anatomical template. Each region is labeled with a leader line; labels include both the traditional anatomical name used by Pappaianni et al. and, in parentheses, the corresponding Larsell numerical lobule designation [144,155]. Panel (**A**) shows a coronal slice through the posterior fossa with the components of the second independent component (IC2) identified by Source-Based Morphometry: the declive (vermal lobule VI), tuber and pyramis (vermal lobules VII–VIII), uvula (vermal lobule IX), inferior semi-lunar lobule (Crus II), and cerebellar tonsil (hemispheric lobule IX). The inferior semi-lunar lobule and cerebellar tonsil are bilateral structures, each indicated by a single label with leader lines to both hemispheres. The figure is rendered in greyscale, and the regions are distinguished from one another by hatch pattern rather than by colour: diagonal hatching, cross-hatching, and stippling of differing density are used solely to separate adjacent regions visually and carry no quantitative meaning. Each region is identified by its leader-line label rather than by its fill. Panel (**B**) shows an inferior axial slice of the cerebrum with the right fusiform gyrus marked with a diagonal hatch pattern, a small cortical region also implicated in the IC2 component. The IC2 component differed significantly between 43 male children with ASD and 46 male controls aged 6 to 12 years (*p* = 0.049). L = left hemisphere; R = right hemisphere.

Among the more replicated structural findings in autism is reduced size of vermal lobules VI and VII. The cerebellar subdivisions referred to throughout this section, including the vermal lobules and their hemispheric extensions, are labeled in Figure 3. The original report by Courchesne, Yeung-Courchesne, Press, Hesselink, and Jernigan [20], based on midsagittal magnetic resonance imaging of the cerebellar vermis in patients with autism, identified significant hypoplasia specifically affecting the neocerebellar lobules VI and VII. The adjacent vermal lobules I–V, which are ontogenetically and phylogenetically distinct, were of normal size, suggesting a region-specific developmental disruption rather than generalized cerebellar atrophy. The authors interpreted this pattern as developmental hypoplasia present from early life rather than postnatal degeneration. Independent laboratories subsequently reported convergent findings: Hashimoto et al. [63] confirmed reduced posterior vermal area in a Japanese cohort of children and adolescents with autism, J. G. Levitt et al. [65] replicated the posterior-vermal reduction in a separate child sample and extended the analysis to additional cerebellar regions, Kaufmann et al. [64] reported vermal lobule VI–VII reduction in a sample combining low-functioning and high-functioning individuals with autism with associated correlations to cognitive measures, Sparks et al. [148] reported cerebellar enlargement in 3- to 4-year-old children with autism that paralleled the broader pattern of early brain overgrowth, and Webb et al. [149] documented atypical cerebellar growth trajectories in toddlers with autism. Postmortem evidence has converged with the imaging findings: Bauman and Kemper [57] and Kemper and Bauman [150] reported reduced Purkinje cell number across the cerebellar hemispheres and posterior vermis in postmortem brains from individuals with autism, with the cell-loss pattern most pronounced in posterior cerebellar regions; Ritvo et al. [151] reported similar Purkinje cell reductions in an independent postmortem series; and Whitney, Kemper, Bauman, Rosene, and Blatt [152] and Whitney, Kemper, Rosene, Bauman, and Blatt [153] extended these findings with stereological methods to document Purkinje cell reductions in additional postmortem cases. While individual studies have varied in the specific lobule emphasized and in the magnitude of effect reported, the cumulative pattern across imaging, postmortem, and meta-analytic work [32] supports posterior vermal abnormality as a robust feature of autistic disorder, with substantial heterogeneity in effect size that the present manuscript attributes in part to diagnostic case-mixing across the four-decade arc. Fetit, Hillary, Price, and Lawrie [154], in the first systematic review to assemble the entire post-mortem literature on autism and related disorders, reported that the cerebellum and the frontal cortex are the two regions most consistently implicated, sometimes with distinct region-specific alterations, while also noting that this literature consists of a small body of studies with small samples. Both observations bear on the present account. The pairing of cerebellar and frontal findings as the most reproducible neuropathological signature is what the combined cerebellar-and-cortical formulation developed below would predict, and the small-sample character of the literature places a ceiling on the precision with which any single lobule-level claim can be established from post-mortem data alone.

Subsequent work from the same laboratory [21] extended these findings in a larger sample of 50 patients with infantile autism aged 2 to 40 years compared with 53 healthy controls. The analysis identified two subtypes: a hypoplasia subgroup comprising approximately 86% of patients, with vermal lobules VI and VII approximately 16% smaller than controls, and a smaller hyperplasia subgroup (12%) with these lobules approximately 34% larger than controls. The bimodal distribution is notable for the present account in two respects. First, it suggests that the cascade may be initiated by different perturbations of cerebellar growth regulation, with both deficient and excessive Purkinje cell or granule cell development potentially yielding altered output through the deep cerebellar nuclei. Second, the bidirectional pattern recurs in conditions discussed below (notably Williams syndrome and, at the level of functional connectivity, ADHD), suggesting that what is critical for the substrate is deviation from typical vermal development rather than the specific direction of deviation. Whether the hyperplasia subgroup represents a mechanistically distinct route to a similar functional endpoint or a variant on a shared developmental dysregulation remains an open question, and is taken up again in the cross-clinical comparison and synthesis sections below. An animal model of the hyperplastic pattern has since become available. Kiffmeyer et al. [156] characterized the cerebellum of the BTBR inbred mouse strain and reported considerable expansion of the cerebellar vermis with abnormal foliation, together with a slight reduction in Purkinje cell density and a marked reduction in Purkinje cell dendritic spine density, in both male and female animals; both sexes showed motor coordination deficits, while only males showed impaired delay eyeblink conditioning. The authors propose that BTBR phenocopies the subpopulation of individuals with ASD who show a hypertrophic cerebellum. The model is informative for the present account because it demonstrates that vermal enlargement can coexist with reduced Purkinje output at the level of dendritic spines, which is the functional convergence that the bidirectional-deviation argument requires: enlargement and reduction in vermal tissue may both compromise Purkinje inhibition of the deep cerebellar nuclei. The correspondence is not exact, because the BTBR expansion falls most heavily on anterior rather than posterior vermal lobules, so the model speaks to the direction-independence of the deviation rather than to the lobule specificity claimed here. Murakami, Courchesne, Press, Yeung-Courchesne, and Hesselink [22] demonstrated that reduced cerebellar hemisphere size accompanied the vermal hypoplasia in some patients, with the cumulative cerebellar slice area correlating positively with the area of vermal lobules VI–VII in the autistic subjects. These findings, derived from samples selected under DSM-III and DSM-IV criteria for autistic disorder, predominantly characterized individuals with the language onset delay and atypical language features that distinguished autistic disorder from related conditions.

Lincoln, Courchesne, Allen, Hanson, and Ene [33] reported a contrasting pattern in seven case studies of individuals with Asperger syndrome. Unlike the consistent vermal VI–VII hypoplasia observed in autistic disorder samples, the Asperger cases showed a more variable pattern, with several individuals demonstrating essentially normal vermal volumes and the group as a whole not demonstrating the reduction characteristic of autistic disorder. Combined with the typically intact early language development that distinguished Asperger syndrome from autistic disorder under DSM-IV criteria, this pattern suggested that vermal lobule VI–VII hypoplasia might be most closely linked to the early language and cognitive features specific to autistic disorder rather than to the broader social and behavioral features common across the autism spectrum. The Lincoln et al. [33] sample is small (*n* = 7), and the anatomical case for retaining the autistic-disorder/Asperger distinction rests on additional convergent evidence rather than this study alone. Direct comparative work distinguishing Asperger syndrome from autistic disorder at the cerebellar level has remained limited under DSM-5 nomenclature, which is itself part of the case-mixing concern motivating the present account. Under DSM-5, this potential subtype distinction is no longer captured at the diagnostic level, although the underlying biological heterogeneity remains. Subsequent volumetric and meta-analytic work has continued to support vermal VI–VII reduction as a robust if heterogeneous feature of autism, with effect sizes varying as a function of sample composition, age, and diagnostic criteria [32,157]. Lobule-level segmentation of a contemporary multisite sample supports the regional specificity of this reduction. Lu et al. [158], applying automated cerebellar lobule segmentation to 75 individuals with ASD and 97 typically developing participants drawn from the Autism Brain Imaging Data Exchange, found lower normalized cortical thickness in the ASD group that was most pronounced in left lobule VI, left Crus I, left lobule X, right lobule VI, and right Crus I. Two aspects are notable. The regions implicated are lobule VI and Crus I, the latter being the hemispheric extension of lobule VII, which is the pairing this review identifies as the phylogenetically recent, prefrontal-projecting territory. Further, reduced thickness in left Crus I correlated positively with the Autism Diagnostic Interview, Revised subscore indexing abnormality of development evident at or before 36 months, tying the cerebellar measure to early developmental deviation rather than to concurrent symptom severity alone. Because the analysis was conducted on ABIDE data without stratification by DSM-IV subtype or language onset, it also indicates that lobule-level measurement can recover regional effects in a mixed contemporary cohort in which whole-vermis volumetric comparisons have often been null.

#### 2.2.6. Vermal Hypoplasia Across Other Clinical Populations

Reduced vermal volume, including but not limited to lobules VI and VII, has been reported across several other developmental and psychiatric conditions, although the specific pattern and lobule involvement vary in ways that may help distinguish among conditions. None of the syndromic conditions discussed in this section is classified as autism spectrum disorder under DSM-5. Each is a distinct genetic, malformative, or developmental diagnosis that may, in some affected individuals, co-occur with an independently established ASD diagnosis (under the DSM-5 specifier “associated with a known medical or genetic condition”). The cross-condition comparison developed in this section is therefore one of conditions that share cerebellar substrate disruption while having distinct primary diagnoses and varying rates of co-occurring ASD when ASD criteria are independently assessed. Joubert syndrome is characterized by congenital cerebellar vermal hypoplasia and aplasia of associated brainstem structures producing the pathognomonic molar tooth sign on axial imaging, and approximately 13% to 27% of children with Joubert syndrome meet criteria for co-occurring ASD [159,160]. Dandy–Walker malformation, defined by vermis hypoplasia, rotation of the vermis away from the brainstem, and an enlarged posterior fossa, is associated with developmental delay and elevated rates of co-occurring ASD [161]. Wells et al. [162] have since placed these figures on a quantitative footing. In a systematic review and meta-analysis of 32 studies comprising 1032 individuals with radiologically confirmed cerebellar malformations, of whom 79% had a malformation involving the vermis, the pooled random-effects prevalence of ASD was 31.2% (95% CI 20.2% to 44.7%), roughly ten times the general population rate, falling to 21.4% after trim-and-fill adjustment for publication bias, with high between-study heterogeneity (I2 = 72.3%). Study-level meta-regression indicated that studies including Joubert syndrome reported lower ASD prevalence (13.9%) than studies without it (38.7%), closely consistent with the 13% to 27% range reported in the earlier Joubert-specific series, whereas the presence of vermis hypoplasia or cerebellar hypoplasia did not significantly moderate prevalence. Two features of this result matter for the present framework. First, it establishes that cerebellar malformation substantially elevates ASD risk while still leaving the majority of affected individuals without an ASD diagnosis, which is the pattern the developmental-window account predicts and which a direct cerebellum-causes-autism account does not. Second, Wells et al. independently arrive at the interpretation advanced here, noting that because several of the variants that produce cerebellar malformation (EN2, RELN, PTEN, TSC1 and TSC2) independently elevate ASD risk, the malformation may be one sign of a broader disruption of brain development rather than the direct cause of the autistic phenotype.

Two features of that meta-analysis require comment, because each bears on the account developed here. The first concerns the study-level subtype analyses, which tested whether the presence of vermis hypoplasia or of cerebellar hypoplasia in a given study predicted the ASD prevalence that study reported. Those analyses proved uninformative, for a reason that is structural rather than substantive. Because 79% of the pooled sample had a malformation involving the vermis, nearly every included study scores positive on the vermis indicator, which leaves little between-study variance for a binary moderator to express. The authors note the more general limitation themselves: meta-regression compares studies to studies rather than individuals to individuals, so a study-level indicator cannot detect a within-sample association between vermal involvement and phenotype. What the analysis does establish descriptively is that the large majority of the autism cases contributing to the pooled prevalence estimate came from individuals whose malformation involved the vermis. The subtype analyses therefore neither support nor undermine a claim about vermal specificity. This is the same interpretive point developed earlier in this review with respect to group statistics computed over compositionally constrained samples: where the composition of the sample restricts the range of the variable being tested, the resulting statistic describes the sample rather than the biology.

The second feature is directly supportive. The substantial heterogeneity reported in that analysis, with an I2 of 72.3%, is attributed by its authors largely to ascertainment. Only 6 of the 32 included studies used any psychometric instrument to support the autism diagnosis, covering 8.5% of the 1032 subjects, with the remainder relying on clinician report or not specifying the diagnostic method. The authors further note that different editions of the DSM, handling autism differently, were in force across the period the studies span, and that these differences may have produced under-identification of autism in the earlier studies conducted under earlier criteria. They also report that intelligence was not consistently assessed, precluding conclusions about the contribution of co-occurring intellectual disability. These are the same three sources of variability that the present review identifies as confounding the cerebellar imaging literature: instrument-based versus impressionistic diagnosis, shifting diagnostic criteria across the span of a literature, and unmeasured cognitive ability. That an independent group arrived at them while addressing a different question supports treating them as general features of this field rather than as a post hoc account of particular findings.

The same authors raise an interpretive alternative that bears directly on the causal structure proposed here. Observing that variants in EN2, RELN, PTEN, and TSC1 and TSC2 affect cerebellar development and independently increase autism risk, they note that in such cases both the malformation and the autism phenotype may reflect a single underlying disruption of brain development, so that the malformation is one sign of that disruption rather than a cause of the phenotype. This is a common-cause account, and it is the most serious rival to the developmental cascade advanced in this review. The two are not mutually exclusive, and the present framework does not require that cerebellar deviation be the sole causal route to the cortical phenotype. It requires only that cerebellar deviation make an independent downstream contribution.

The accounts are nonetheless distinguishable in principle, and stating how is the appropriate response to the alternative. A pure common-cause account predicts that the cortical connectivity signature should appear in cases carrying the relevant variant irrespective of whether cerebellar development was in fact perturbed, and should be absent where cerebellar perturbation arises from a cause that does not independently affect cortex. The cascade account predicts the reverse, that the signature should track the cerebellar perturbation rather than its etiology. The informative cases are therefore those in which cerebellar deviation arises environmentally rather than from a variant with independent cortical effects, among them fetal alcohol spectrum and prenatal valproic acid exposure, where the cascade account predicts that the cortical signature should be present and the common-cause account does not. Neither prediction has been tested directly. The genetic syndromes reviewed in this section are for that reason the least evidentially decisive of the cross-condition comparisons, whatever their heuristic value, and the environmental exposures are the more informative test cases despite having received less attention.

#### 2.2.7. A Skeptical Case, and What It Constrains

A dissenting position deserves direct treatment. Baizer [163] has questioned whether cerebellar dysfunction has the causal role commonly attributed to it in autism, on three grounds. Cerebellar damage does not consistently produce autism. Cerebellar damage acquired in adulthood does not produce the behavioral profile seen in children with early cerebellar injury. And autism is not observed in every condition involving reduced Purkinje cell numbers. Each observation is correct, and each is worth stating as a constraint on any cerebellar account rather than as an objection to be dismissed.

The framework developed here is compatible with all three observations, and the second is not merely compatible but predicted. The account is neurodevelopmental in a specific sense that the objection does not engage. What the cerebellum contributes during the sensitive period is the scaffolding of cortical predictive models while those models are being constructed. Once constructed, predictive templates are instantiated in cortical connectivity and persist. Cerebellar damage acquired in adulthood therefore degrades the online operation of predictive models that already exist; it does not prevent their formation, because formation is complete. These are categorically different consequences, and expecting them to converge on the same phenotype misconstrues what a developmental account claims. The adult cerebellar patient has intact templates and impaired execution. The child with early cerebellar disruption has templates that were never adequately built, which is why the resulting profile is developmental rather than a motor or coordination syndrome. Autism is a disorder of the construction of these systems, not of their subsequent operation in an otherwise normally developed brain.

The same reasoning applies with equal force to the Purkinje cell argument, and it sharpens what the framework actually requires. Purkinje cell number as such is not the operative variable. What matters is the developmental interval in which the reduction is expressed. The account requires that the reduction be prenatal in origin, or expressed within approximately the first two postnatal years, while cortical networks are still being organized and pruned. Purkinje loss arising after that interval, whether through adult-onset degeneration or in mutant lines whose cell loss occurs late, falls outside the scaffolding period and is not predicted to produce the autistic phenotype. The available evidence places the loss in autism within the relevant window rather than outside it: postmortem findings indicate reduced Purkinje cell number without corresponding evidence of reduced production, and in the mutant lines that model autism-like phenotypes the reduction typically arises through early postnatal degeneration rather than in maturity. That conditions with late-onset Purkinje loss do not produce autism is therefore not a counterexample to the account. It is a specific consequence of it.

What the skeptical case does establish, and what should be conceded plainly, is that a cerebellar account cannot be a general theory of autism. The heterogeneity of the phenotype makes it implausible that any single structure explains all of it, and dysfunction originating outside the cerebellum most likely accounts for a substantial share of cases, particularly those whose presentation does not include the early language and contextual features this review has treated as the focal phenotype. The systemic features frequently reported in autism, including circadian disruption, immune dysregulation, and gastrointestinal alteration, are not addressed by this framework at all. The claim advanced here is correspondingly bounded: that within the language-delayed autistic disorder phenotype, in which the vermal findings were established, the cerebellar contribution is causal rather than incidental, and the developmental cascade specifies how. Whether that phenotype constitutes a large or small proportion of the contemporary spectrum is an empirical question that the stratification analyses proposed throughout this review are designed to answer.

Fragile X syndrome, the most common inherited cause of intellectual disability, results from FMR1 silencing with loss of fragile X mental retardation protein (FMRP), and approximately 30% of affected males meet criteria for co-occurring ASD (the condition is not itself an ASD diagnosis but is one of the most frequently identified syndromic causes of co-occurring autism). Fragile X has been associated with reduced posterior vermal volume that correlates with cognitive impairment and social cognition difficulties [164,165]. Chen et al. [166] reviewed the contemporary evidence on cerebellar neuropathology and motor skill deficits in fragile X syndrome, and Li et al. [167], comparing 150 children across fragile X, idiopathic ASD, and typically developing groups, found that both clinical groups showed decreased functional connectivity within the cerebellum network and between that network and the default mode, sensorimotor, and visual networks, with aberrant topological alterations of the cerebellum network in both; connectivity within the default mode network was reduced in fragile X specifically. Critically for the cross-condition argument, the correlations between social affect severity and mean cerebral–cerebellar connectivity followed significantly different trends in fragile X than in ASD, and in the fragile X group a topological measure at cerebellar Crus I was negatively associated with DNA methylation level. Feng et al. [168] extended this to structure in an overlapping cohort, reporting that children with fragile X showed increased gray matter volume in caudate and cerebellar Crus I but decreased volume in frontal insular regions and in cerebellar vermis lobules VIII and IX, a lobule-level profile distinct from the vermis VI–VII reduction characteristic of autistic disorder, while the ASD group showed faster gray matter growth rates. The two studies together indicate that clinically overlapping conditions can be separated at the level of which cerebellar subregion is affected, which is the resolution at which the present framework makes its claims. FMRP loss simultaneously disrupts cerebellar substrates and cortical mGluR5-dependent synaptic plasticity required for context updating, providing a clear example of a single molecular cause producing combined cerebellar-and-cortical disruption at relevant developmental windows. Attention-deficit/hyperactivity disorder, while less consistently associated with cerebellar abnormalities than ASD, has shown reductions in posterior cerebellar vermis volume in several studies, with associations to inhibitory control and timing functions [169,170]. Rett syndrome, caused by MECP2 dysfunction and characterized by developmental regression, shows cerebellar and vermal atrophy alongside cortical and subcortical alterations [171]. Under DSM-IV-TR, Rett’s disorder was classified among the pervasive developmental disorders. DSM-5 removed it from the autism spectrum because the molecular etiology is known and because the phenotype, while sharing surface features with ASD during the regression phase, has a distinct neurobiological cause and course. MECP2 dysfunction similarly affects multiple substrates simultaneously, with the Rett phenotype reflecting the combined failure rather than a downstream consequence of cerebellar involvement alone. Prader–Willi syndrome, a 15q11-q13 imprinting disorder, has shown altered functional network architecture with enhanced dorsolateral prefrontal functional connectivity and attenuated frontopolar–parietotemporal connectivity relative to typically developing controls, with Autism-Spectrum Quotient scores correlating positively with right frontal connectivity [172]; a subset of affected individuals meet criteria for co-occurring ASD.

Schizophrenia presents a particularly informative contrast. J. J. Levitt and colleagues [173], in work prompted by the Courchesne et al. [20] report in autism, measured vermal lobule areas in 30 male patients with schizophrenia. This is a different research group from the J. G. Levitt team whose autism findings are described above, and the two 1999 reports are distinguished here by author initials throughout. Contrary to the autism pattern, they reported larger cerebellar structures overall in schizophrenia, with patients without perinatal injury showing larger lobules VI–VII than controls. This finding suggests that vermal lobule VI–VII alterations are not a non-specific marker of psychiatric disturbance but follow distinct, disorder-specific patterns. Fetal alcohol spectrum disorder, in contrast to autism, has been associated more prominently with reduction in anterior vermal lobules I–V rather than the posterior lobules VI–VII [174], providing a further dissociation across developmental etiologies. Kallmann syndrome, a rare disorder of gonadotropin-releasing hormone neuron migration, has been linked in case series to partial cerebellar vermis hypoplasia [175,176]. The 3q29 deletion syndrome, which confers a 40- to 100-fold increased risk for schizophrenia alongside elevated risk for autism and intellectual disability, provides an especially informative recent case because it spans both poles of the severe mental illness frame adopted in this review. Sefik et al. [177], comparing 24 individuals carrying the deletion with 1608 neurotypical controls on structural MRI, reported smaller cerebellar cortex volumes both before and after correction for intracranial volume, with an anterior–posterior gradient emerging in lobule-based and voxel-wise analyses, together with larger cerebellar white matter volumes and elevated rates of posterior fossa arachnoid cysts and mega cisterna magna. Cerebellar white matter and subregional gray matter volumes were associated with visual perception, visual–motor integration, and IQ measures, whereas the cystic malformations carried no behavioral association. Two features bear on the present framework. The emergence of an anterior–posterior gradient supports the regional rather than global character of the cerebellar involvement argued for here, although the behavioral correlates reported were visuomotor and general-cognitive rather than the language and social measures central to the autistic-disorder phenotype. The authors also conclude that cerebellar pathology constitutes an intersection point between syndromic and idiopathic forms of neurodevelopmental disability, which is the cross-condition position developed in this section. Moreno-Rius [178] articulated the inferential rationale for comparisons of this kind, observing that because individuals with ASD present with both social and motor deficits, cerebellar findings within ASD alone cannot establish a cerebellar contribution to social behavior specifically, and that disorders whose principal symptom is disordered social behavior are therefore necessary comparators. The same logic underwrites the cross-condition strategy adopted throughout this section.

Williams syndrome occupies a particularly informative position within this comparative picture, because the cerebellar finding runs in the opposite direction from that observed in autistic disorder while the cognitive phenotype shares a notable feature. Williams syndrome is caused by a hemizygous deletion of approximately 25 to 28 contiguous genes on chromosome 7q11.23, including LIMK1, CLIP2, GTF2I, GTF2IRD1, and ELN, none of which are deleted in autism. Williams syndrome is not classified as an ASD subtype under DSM-5, and co-occurring ASD diagnoses in Williams populations are relatively uncommon (approximately 7% to 12% in published series), reflecting the contrast between the hypersocial Williams phenotype and the reduced social engagement that characterizes the classical ASD presentation. Niego and Benitez-Burraco [179] reported that autism and Williams syndrome, despite their dissimilar socio-cognitive profiles, show overlapping patterns of abnormal gene expression in peripheral blood. Because this is a peripheral rather than a central measure, it is offered only as suggestive of shared molecular involvement across the two conditions, and not as evidence about cerebellar or cortical gene expression. With that qualification, it is consistent with the present framework’s claim that a shared substrate-level perturbation can yield opposite surface phenotypes depending on the cortical genetic and developmental context within which it occurs. Within this distinct cortical genetic context, neuroimaging studies have consistently reported relative enlargement of the cerebellar vermis, particularly of lobules VI and VII, in Williams syndrome compared with typically developing controls [180,181,182], even after adjustment for the overall reduction in total brain volume characteristic of the syndrome. This pattern is structurally parallel to the approximately 12% hyperplastic subgroup identified within the Courchesne et al. [21] autistic-disorder sample. The presence of motor signs in Williams syndrome (hypotonia, gait abnormalities, joint laxity, and visuomotor integration deficits) provides independent evidence that the enlarged vermis is dysregulated rather than functionally enhanced, since vermal enlargement is not associated with superior motor or cognitive performance in this population. The behavioral phenotype differs from autistic disorder in the direction of social engagement, with hypersociability and indiscriminate friendliness characterizing Williams syndrome and reduced social engagement characterizing autistic disorder. The deeper commonality is a failure of context-dependent social inference. Individuals with Williams syndrome misread social context by approaching strangers as if they were intimates, by failing to modulate emotional intimacy to relational distance, and by missing contextually inferred meanings that would normally limit social approach. Individuals with autistic disorder misread social context differently, by withdrawing in situations that call for engagement or by engaging in ways that miss the contextual frame. The content of the social–cognitive failure differs; the form of the failure is similar, with both populations showing impaired integration of social cues across context, social-role inference, and prediction of social meaning. The dissociation observed by Lincoln, Lai, and Jones [133] of shifting and joint attention in Williams syndrome supports a cerebellar contribution to social attention deficits across both directions of vermal deviation. Intellectual disability is variably present in both syndromes, with a subset of individuals in each population showing relatively spared general cognitive capacity alongside the context-inference deficit, indicating that the cerebellar-cortical disruption of context-dependent inference is dissociable from generalized cognitive impairment.

Together, these comparative findings indicate that posterior vermal abnormality, while not exclusive to autism, is most consistently and characteristically associated with autism among the conditions in which it has been studied, and that vermal deviation from typical development in either direction may compromise cerebellar predictive scaffolding of cortical associational networks supporting context-dependent inference. Table 2 organizes the structural and connectivity findings reviewed here by severe mental illness subtype, with autistic disorder placed first as the prototype case against which the comparison conditions are read. It records for each study the method, the sample and the diagnostic frame under which it was ascertained, the principal finding, and whether cerebellar and connectivity abnormalities were identified. Read in that order, the table shows that cerebellar involvement is transdiagnostic while the vermal lobule VI and VII signature is not. The contrast with schizophrenia (vermal enlargement in non-perinatally injured cases) and with fetal alcohol spectrum disorder (anterior rather than posterior vermal reduction) supports the view that vermal volumetric measures carry diagnostic information rather than reflecting generic neurodevelopmental disturbance. A further parallel holds between the 12% hyperplastic subgroup within autistic disorder [21] and the vermal enlargement of Williams syndrome [180,181]. It suggests that what matters for the autistic-disorder phenotype and its cognate context-inference failures is deviation from typical vermal development rather than the direction of that deviation. The cortical genetic and developmental context within which the cerebellar abnormality occurs then determines the surface phenotype. The selective implication of phylogenetically recent, prefrontal-projecting vermal regions is consistent with the broader pattern of altered cerebellar network organization reported in functional and structural connectivity studies [29,31,131] and converges with the transdiagnostic cerebellar connectivity findings reviewed in the following section. At the network-connectivity level, recent large-cohort transdiagnostic analyses are also informative. Fu, Sui et al. [183] reported, across two independent samples (*n* = 311 schizophrenia + healthy control; *n* = 569 autism + healthy control), increased dynamic functional network reconfiguration between cerebellar and subcortical/sensorimotor domains shared by autistic disorder and schizophrenia. Du et al. [184], assembling approximately 600 individuals with schizophrenia, 1000 with ASD, and 1700 healthy controls, reported that both conditions showed lower functional integration within default mode and sensorimotor domains and increased interaction between cognitive control and default mode domains, and, structurally, reduced gray matter volume and density in the occipital gyrus and the cerebellum in both illnesses, with the ASD group showing weaker changes than the schizophrenia group across the shared abnormalities while interactions between visual and cognitive regions carried the disorder-unique deficits. Diao et al. [185], in the largest such analysis to date (*n* = 2176; autism, attention-deficit/hyperactivity disorder, schizophrenia, and healthy controls), identified a shared transdiagnostic abnormal connectivity pattern linking the cerebellum, brain stem, and subcortical network to cortical perceptual-executive networks, with autism and attention-deficit/hyperactivity disorder showing connectivity deviations in the same networks but in opposite directions (decreased in autism, increased in attention-deficit/hyperactivity disorder). The Diao et al. analysis operated at the level of whole-brain functional networks rather than at the level of specific vermal lobules, and the framework employed was a transcriptomic-and-neurotransmitter annotation rather than a predictive-modeling one; the convergence with the present account is therefore at the level of the network-connectivity finding (cerebellum participating in a transdiagnostic abnormal connectivity pattern, with bidirectional deviations) rather than at the level of specific vermal anatomy or predictive-coding mechanism. With those qualifications noted, the bidirectional cerebellar-cortical network finding in Diao et al. [185] converges with the bidirectional vermal-structural finding of Courchesne et al. [21] and with the Williams syndrome parallel discussed earlier, supporting the more general framework on which the present account rests.

The cross-condition pattern reviewed above also raises a more general consideration that refines the cascade model developed in the following section. Vermal hypoplasia is observed in conditions whose molecular causes (FMR1 silencing in fragile X, MECP2 dysfunction in Rett, the 7q11.23 deletion in Williams, the 15q11-q13 imprinting defect in Prader–Willi, FOXC1 disruption in some FOXC1-related Dandy–Walker, AHI1 mutations in some Joubert cases) affect multiple brain regions simultaneously rather than producing cerebellar damage in isolation. Context-dependent inference deficits in these conditions are accordingly best understood not as downstream consequences of vermal hypoplasia alone but as arising from the combined disruption of cerebellar substrates and cortical context-integration networks at developmental windows when both are vulnerable. The prenatal window during which vermal lobules VI–VII are forming coincides with critical periods for thalamocortical relay neuron migration, cortical layer specification in association cortex, and the early phases of cortical synaptic exuberance. A perturbation occurring during this window may plausibly affect any or all of these substrates simultaneously. On this temporal-coincidence reading, vermal hypoplasia in the conditions reviewed serves as a developmental-window marker that the upstream perturbation reached a stage at which posterior cerebellar tissue was vulnerable, rather than as a sufficient cause of the cognitive phenotype. The autistic-disorder phenotype is then best understood as one specific cluster that emerges when an upstream perturbation hits a developmental window that catches both cerebellar context-prediction substrates and the cortical context-integration substrates they scaffold. The cascade model developed in the following section is one specific instantiation of this more general principle; it specifies the pathway through which cerebellar disruption alone could contribute to the cortical phenotype, while recognizing that in many cases the upstream perturbation acts on cerebellar and cortical substrates concurrently.

#### 2.2.8. The Psychotic Spectrum as a Test of Generalization

If the cerebellar account developed here is an account of a developmental window rather than of a single diagnosis, the psychotic spectrum disorders are the case on which it should be tested most severely. Schizophrenia is the condition within the severe mental illness class whose clinical onset is furthest removed from the prenatal period in which the vermal deviation is proposed to arise, and it is therefore the case in which a purely coincidental cerebellar finding is most likely to be exposed. It is also the condition for which the neurodevelopmental hypothesis is best developed independently of the autism literature [1,2,3], which means the prediction can be evaluated against an existing body of evidence rather than constructed post hoc.

Cerebellar involvement in schizophrenia is well documented at the level of the structure as a whole. Andreasen and Pierson [188] reviewed the accumulated structural, functional, and connectivity evidence and argued that cerebellar dysfunction is central rather than incidental to the disorder, proposing that disrupted cortico-cerebellar-thalamic-cortical circuitry produces the impairment in the timing and coordination of mental activity that characterizes the psychotic state. Moberget et al. [189], in a multisite mega-analysis of 983 patients and 1349 healthy controls, reported reduced cerebellar volume in schizophrenia together with altered cerebello-cerebral structural covariance, establishing the cerebellar finding at a sample size that the autism literature has not matched for any single structural measure. The scale of that analysis matters for the present argument, because the replication problem this review is centrally concerned with is a problem of statistical power interacting with cohort composition, and the schizophrenia literature has largely escaped the diagnostic-drift confound that complicates the autism findings.

The regional and directional pattern is where the comparison becomes informative, and it is not uniform. J. J. Levitt and colleagues [173] reported vermal lobules VI and VII larger than controls in patients without perinatal injury, which is the opposite direction from the autistic disorder finding at the same landmark. Moberget and colleagues reported reduced total cerebellar volume rather than a specified lobular territory, and the first-episode evidence reviewed below localizes to a dispersed set of regions. These three results are not straightforwardly reconcilable, and this review does not claim to reconcile them. What they share is that none reproduces the focal posterior vermal reduction that characterizes autistic disorder. The cerebellum is implicated in both conditions, but not in the same way, which is the observation the framework requires and which a generic account of cerebellar involvement in psychopathology would not predict.

What the contrast does establish is the point the framework requires. Posterior vermal deviation is not a generic marker of psychiatric disturbance, because if it were, it would appear in the same direction across conditions, and it does not. It behaves instead as a marker of perturbation within a shared developmental window, with the direction of deviation and the resulting phenotype determined by the cortical context in which the perturbation occurs. The directional heterogeneity documented in the cross-condition section above, in which the same vermal landmark is reduced in most of an autistic disorder sample and enlarged in a minority and in Williams syndrome, is the clearest illustration of this principle.

At the network level, the transdiagnostic evidence is more consistent than the volumetric evidence and converges on shared cerebellar involvement. Fu, Sui, and colleagues (2020) [183] reported, across two independent samples comprising 311 individuals in a schizophrenia and control cohort and 569 in an autism and control cohort, increased dynamic functional network reconfiguration between cerebellar and subcortical or sensorimotor domains common to both conditions. Du et al. [184], assembling approximately 600 individuals with schizophrenia, 1000 with autism spectrum disorder, and 1700 healthy controls, reported lower functional integration within default mode and sensorimotor domains in both conditions together with reduced cerebellar gray matter volume and density, with the autism group showing the weaker effects across the shared abnormalities. Diao et al. [185], in the largest such analysis conducted to date at *n* = 2176 spanning autism, attention-deficit/hyperactivity disorder, and schizophrenia, identified a shared transdiagnostic connectivity abnormality implicating the cerebellum across all three. Hwang et al. [191] reported thalamic connectivity alterations in schizophrenia that parallel those described in autism, consistent with a shared disturbance of the cerebello-thalamo-cortical loop that the cascade model places at the center of the account. Fryer et al. [192] extended that observation backward in the illness course, reporting thalamic dysconnectivity in the psychosis risk syndrome and in early illness schizophrenia, which places the disturbance before the onset of frank psychosis rather than after it. Feng et al. [193] reported altered functional connectivity of cerebellar networks at first episode. Wang et al. [195] reported cerebellar-basal ganglia dysconnectivity related to motivational deficits, extending the affected circuitry beyond the thalamocortical loop. Of this group, Abram et al. [194] bears most directly on the predictive-modeling account developed here: pons-to-cerebellum hypoconnectivity along the psychosis spectrum was associated with impaired sensory prediction and with hallucination severity. Because the pontine nuclei constitute the principal cortical input to the cerebellum, a hypoconnectivity finding at that junction implicates the forward-model machinery itself rather than its downstream targets, and it does so using the same functional currency, sensory prediction, that the cerebellar account of autism invokes.

Three limits on this evidence should be stated plainly, because they constrain what the severe mental illness framing can claim. The first has been narrowed by recent work. Much of the schizophrenia imaging literature is acquired after illness onset and after antipsychotic treatment has begun, so illness duration and medication exposure cannot in general be excluded as contributors. Two findings narrow that concern. Ding et al. [190] restricted a meta-analysis to first-episode drug-naive patients, combining voxel-based morphometry, amplitude of low-frequency fluctuation, and functional connectivity strength across 783 patients and 704 controls, and reported cerebellar abnormality in the absence of any medication exposure. Fryer et al. [192] reported thalamic dysconnectivity in the psychosis risk syndrome, before frank illness. Neither reaches the neonatal and infant evidence that anchors the developmental claim in autism, and the schizophrenia literature still has no equivalent of it. The findings can no longer be attributed wholly to treatment or to chronicity. Second, the transdiagnostic analyses establish shared network abnormality but not shared developmental origin, since convergent adult connectivity patterns are compatible with distinct developmental routes. Third, the divergence among the schizophrenia vermal findings remains unresolved, and no study has measured vermal lobules VI and VII in schizophrenia and in autistic disorder within a single design and a single measurement protocol, so the contrast drawn here rests on comparison across the literature rather than within a study. The claim advanced here is therefore the weaker and more defensible one: the cerebellum is implicated across the severe mental illness class, the implication is regional rather than global, and the developmental cascade is specified in detail only for autistic disorder, where the requisite early evidence exists.

#### 2.2.9. Obsessive–Compulsive Disorder and the Cerebello-Thalamo-Cortical Circuit

Obsessive–compulsive disorder provides a second and narrower comparison. It is included here not because the cerebellar literature in obsessive–compulsive disorder is extensive, and this review makes no attempt to survey it comprehensively, but because the circuit it implicates is the circuit the present cascade model runs on. Sha et al. [196] reported functional disruption of cerebello-thalamo-cortical networks in obsessive–compulsive disorder, placing a fourth condition on the same loop already traced through autistic disorder and the psychotic spectrum. Xu et al. [197] reported structural and functional deficits together with altered structure–function coupling, graded by symptom severity across moderate and severe presentations, which is the kind of dose relationship that argues against the abnormality being incidental. Shobeiri et al. [198], reviewing the diffusion tensor imaging literature systematically, found cerebellar microstructural abnormality reported across studies, extending the finding from function to white matter organization. Yan et al. [199] followed drug-naive patients longitudinally and reported cerebellar functional connectivity changes over the treatment course alongside associated gene expression patterns.

What this comparison contributes is a boundary rather than a convergence. The obsessive–compulsive disorder literature implicates the cerebellum at the circuit level and does not report the focal posterior vermal finding that characterizes autistic disorder, and no claim about vermal lobules VI and VII is advanced for it here. That absence is informative. Together with the dispersed schizophrenia distribution described above and the anterior vermal localization in fetal alcohol spectrum, it indicates that cerebello-thalamo-cortical disturbance is common across severe mental illness while the particular cerebellar territory involved is not. The framework developed in this review predicts exactly that pattern, because it locates the specificity in the timing of the perturbation and in the cortical context receiving the altered cerebellar output rather than in cerebellar involvement as such.

#### 2.2.10. The Case-Mixing Problem Generalizes Across Severe Mental Illness

The diagnostic-drift problem described for autism is not specific to autism, and the comparison conditions reviewed above are not immune to it. The same edition of the Diagnostic and Statistical Manual that collapsed the autism subtypes into a single spectrum diagnosis also eliminated the classical schizophrenia subtypes, removing the paranoid, disorganized, catatonic, undifferentiated, and residual categories on grounds of limited diagnostic stability and poor predictive validity, and replacing them with a single schizophrenia diagnosis situated within a schizophrenia spectrum chapter and supplemented by dimensional severity ratings. That is structurally the same revision: subtypes collapsed into a spectrum, with the distinguishing features relocated to specifiers or to dimensional ratings rather than retained as criteria.

The methodological consequence should therefore generalize. If any cerebellar or connectivity finding in schizophrenia were specific to one of the former subtypes, or to a cohort characteristic that the subtype distinction happened to track, then pooled analyses of spectrum-defined samples ascertained after 2013 would attenuate that finding in the same way that pooled DSM-5 autism samples attenuate the vermal effect. The prediction is symmetric with the one advanced for autism and is equally testable. Findings established in pre-DSM-5 schizophrenia cohorts should replicate less reliably in contemporary spectrum-defined samples, and stratification by the features the former subtypes tracked should recover them. The directionally inconsistent vermal findings reviewed earlier, where enlargement was reported in a single-site sample of thirty patients and reduction in a pooled multisite mega-analysis, are one place where this possibility warrants examination rather than being treated as a straightforward methodological discrepancy.

Stated at the level of the class, the point is this. Wherever a neurobiological finding was established under narrower diagnostic criteria than those now in force, broadening of the criteria will dilute the finding in pooled analyses, and the dilution will be mistaken for failure to replicate. As diagnostic systems across severe mental illness continue to move from categorical toward dimensional and spectrum-based definitions, and as attenuated and risk syndromes are incorporated into research ascertainment, this problem should be expected to recur rather than to resolve. The remedy is not a return to the older categories, which were abandoned for defensible reasons, but the retention of the cohort characteristics they tracked in a form that permits retrospective stratification.

The regional point deserves precision, because it is what the comparison establishes. The distribution reported by Ding and colleagues spans lobules III to VI, VIII, and Crus I and II. That is a dispersed pattern rather than the focal posterior vermal involvement characterizing the autistic disorder findings. Cerebellar involvement in schizophrenia is therefore not a weaker version of the autism finding at the same landmark. It is involvement of different cerebellar territory, which is what a developmental-window account predicts when the perturbation and the cortical context both differ.

#### 2.2.11. A Developmental Cascade Model: Cerebellar Origins of Cortical Connectivity Disruption

The pattern of findings reviewed above, with reduced posterior vermis size co-occurring with posterior cerebral white matter alterations and the characteristic ASD pattern of long-range underconnectivity and local overconnectivity, raises a developmental question: are these alterations parallel and independent consequences of shared upstream causes, or does early cerebellar pathology drive the subsequent cortical phenotype through a developmental cascade? Courchesne and colleagues [69,70,71] proposed the latter framework on the basis of three observations available to them by the early 1990s. First, cerebellar development is largely prenatal, with the cerebellar plate forming in the embryonic period and Purkinje cell migration completed by approximately 20 to 24 weeks gestation, considerably earlier than the protracted postnatal development of cortical association areas [200]. The hypoplasia of vermal lobules VI and VII identified in autism is therefore consistent with a prenatal origin. Second, the cerebellar cortex projects, through Purkinje cell axons, inhibitory input to the deep cerebellar nuclei, which in turn provide excitatory output, via the superior cerebellar peduncle and thalamus, to widespread cortical targets including prefrontal and parietal association areas [55]. Loss or dysfunction of Purkinje cells therefore disinhibits the deep cerebellar nuclei and increases excitatory drive from cerebellum to thalamus to cortex. Third, this disinhibitory cascade reaches the cerebral cortex during the protracted postnatal period when synaptogenesis, synaptic pruning, and myelination shape long-range cortical connectivity, providing a mechanism by which a prenatal cerebellar insult could influence postnatal cortical maturation.

On this account, the frequently reported ASD pattern of local cortical overconnectivity with reduced long-range connectivity [41,44,46,47,49,76,77,78] need not be read solely as a primary cortical defect. It is also interpretable as a downstream consequence of altered cerebellar-thalamic-cortical excitatory tone during sensitive periods of cortical development. Mechanistically, the model predicts that disrupted Purkinje cell inhibition of the deep cerebellar nuclei increases excitatory drive through the cerebello-thalamo-cortical loop. The sensitive window for this effect begins in the third trimester, when Purkinje cell migration is largely complete, and extends through the first two postnatal years, when cortical synaptic density peaks and activity-dependent pruning begins [62,200,201]. Recent work in genetic and circuit-level models has provided convergent support: D’Mello and Stoodley [73] reviewed cerebro-cerebellar circuit alterations in autism with attention to the developmental cascade, Stoodley et al. [75] demonstrated that chemogenetic perturbation of right Crus I in mice produces autism-relevant social and repetitive-behavior phenotypes that are rescued by stimulation of the same region, Kelly, Meng et al. [202] used optogenetic inhibition of cerebellar output to recapitulate autism-relevant behaviors, and Sathyanesan et al. [74] reviewed emerging connections among autism, cerebellar development, and dysregulated cerebellar output. Convergent findings have since accumulated across additional genetic models. Kawamura et al. [203] demonstrated that CHD8, among the most frequently disrupted genes in autism, is required for cerebellar development and motor function, but reported a dissociation of direct relevance to the present argument: granule-neuron-progenitor-specific deletion of Chd8 produced cerebellar hypoplasia and a motor coordination defect without producing ASD-like behavioral abnormalities. Cerebellar hypoplasia arising from a high-penetrance autism risk gene is therefore not by itself sufficient to generate the behavioral phenotype, which is precisely the conclusion the developmental-window formulation of this review requires and which a simple cerebellum-causes-autism account does not predict, and Wang et al. [204] reported that impaired cerebellar plasticity hypersensitizes sensory reflexes in SCN2A-associated autism. A methodological caution applies to this literature as a whole. Otazu et al. [205] reported glio-neuronal heterotopia in both the cerebellar vermis and the neocortex of Shank3 and Cntnap2 mice, but attributed these malformations to the C57BL/6 background strain, on which the models are maintained, rather than to the autism-associated mutations themselves. Because C57BL/6 mice display such malformations spontaneously, cerebellar and cortical abnormalities in genetic mouse models on this background cannot be assumed to be consequences of the targeted gene, and the inferential weight that the mouse literature can carry for a cerebellar-origin account is correspondingly bounded. van der Heijden, Gill, and Sillitoe [187], reviewing abnormal cerebellar development in autism spectrum disorders, note two facts that bear directly on the timing argument advanced here: only 5% to 30% of ASD cases are explained by a known genetic cause, and up to 40% of infants sustaining cerebellar hemorrhages and lesions receive an ASD diagnosis. Such hemorrhages are overrepresented in severely premature infants, that is, in infants whose injury falls within a window of highly dynamic cerebellar development involving an approximately five-fold size expansion and extensive rearrangement of local circuits. This converges with the perinatal-injury evidence of Limperopoulos et al. [206] and supports the proposition that when the cerebellar disruption occurs matters as much as whether it occurs. Duan et al. [207] reviewed the same territory from the direction of motor skill learning, covering Fmr1, Chd8, Shank3, BTBR, 16p11.2, and Mecp2 models together with the cerebellar and cerebral regions and connectivity pathways implicated in each, and so offers a consolidated entry point to the several models cited separately across this review. Within this window, elevated thalamocortical excitatory tone would favor strengthening of locally clustered synapses available at the time of perturbation, while disrupting the slower activity-dependent processes that establish and refine long-distance association tracts [42,208]. The model further predicts greatest structural impact on the most ontogenetically late-maturing tracts, with the corpus callosum, which carries interhemispheric projections from cortical areas that mature on a protracted postnatal schedule, a particular candidate. Consistent with this prediction, multiple independent laboratories have reported reduced corpus callosum size in autism, with effects most prominent in posterior and middle callosal regions carrying parietal and temporo-occipital fibers [24,79,80,81,82]. Saitoh, Courchesne, Egaas, Lincoln, and Schreibman [209] extended these findings by demonstrating that, in a sample of 33 individuals with autism, the regions showing significant volumetric reduction were vermal lobules VI and VII and the posterior corpus callosum (each more than 9.9% smaller than in controls), with the volumetric reductions in these two regions correlating significantly with one another. Diffusion-imaging follow-ups have converged on reduced callosal microstructural integrity [4,210], consistent with the structural cascade model.

The pattern warrants more precise statement than a single phrase allows, because the underlying literature is neither uniform nor uniformly interpreted. On the local side, Keown, Shih, Nair, Peterson, Mulvey, and Muller [76], using resting-state functional magnetic resonance imaging with graph-theoretical analysis, reported increased local functional connectivity in bilateral temporo-occipital regions in adolescents with ASD, with greater posterior overconnectivity associated with greater symptom severity. Maximo, Keown, Nair, and Muller [211], examining 29 participants with ASD and 29 typically developing participants across multiple measures and spatial scales, found local overconnectivity in occipital and posterior temporal regions accompanied by local underconnectivity in cingulate and medial prefrontal regions, so that the local finding is regionally specific rather than global. On the long-range side, Just, Cherkassky, Keller, Kana, and Minshew [46] provided both functional and anatomical evidence of reduced connectivity during an executive function task, together with corpus callosum morphometry, which is the pairing on which the cortical underconnectivity account principally rests.

Network-level analyses complicate the simple two-term description further. Rudie et al. [77] found reduced short-range and long-range connectivity within functional systems but stronger connectivity between them, yielding reduced modularity and clustering alongside shorter characteristic path lengths and higher global efficiency. That is a loss of functional segregation rather than a loss of connectivity as such, and it is not well captured by describing the phenotype as underconnected. Supekar et al. [78] reported the opposite direction outright, finding greater functional connectivity in children with ASD at both whole-brain and subsystem levels, across short-range and long-range connections, with greater connectivity associated with greater social impairment.

Three qualifications follow, and the present framework should be read as constrained by them. First, the divergence across studies is partly methodological. Muller, Shih, Keehn, Deyoe, Leyden, and Shukla [49], surveying 32 functional connectivity studies, found that reports supporting generalized underconnectivity differed systematically from those that did not in filtering, task regression, and whole-brain versus region-of-interest approaches, and concluded that both reduced connectivity and diffusely increased connectivity may reflect aspects of network dysfunction in ASD. Second, the local findings vary by site and by acquisition condition. Nair, Jao Keehn, Berkebile, Maximo, Witkowska, and Muller [212], across 147 participants with ASD and 184 typically developing participants, found a broadly consistent pattern of local overconnectivity in posterior and visual regions in eyes-open datasets alongside local underconnectivity in cingulate regions, with voxel-level findings varying across samples and eyes-closed data producing a different pattern. Third, local connectivity as measured with functional magnetic resonance imaging is not equivalent to microscopic or minicolumnar connectivity, and the two should not be treated as interchangeable evidence for the same claim.

The cascade advanced here does not require that the phenotype be uniformly underconnected at long range. What it requires, and what the evidence above supports across otherwise divergent studies, is a systematic disturbance in the balance between local and distributed processing, expressed as reduced functional segregation among cortical systems. Whether a given study reports that disturbance as overconnectivity or as underconnectivity depends substantially on the measure, the spatial scale, and the processing pipeline. Stating the claim at the level of segregation rather than of connection strength makes it compatible with the full range of reported findings without weakening it, and it is the level at which altered cerebello-thalamo-cortical drive during cortical network formation would be expected to have its effect.

#### 2.2.12. Molecular and Synaptic Substrates of the Cascade

The developmental cascade framework gains additional support from work on neurogenetic disorders. Tsai et al. [213] demonstrated in a mouse model with selective Purkinje cell deletion of Tsc1 that targeted cerebellar pathology produces ASD-like social, communicative, and repetitive behavioral phenotypes alongside altered cortical connectivity, providing direct experimental evidence that a primary cerebellar lesion can generate an autism-like syndrome. Wang, Kloth, and Badura [62] synthesized the developmental neuroscience literature in proposing that the cerebellum guides the maturation of remote nonmotor neural circuitry through a sensitive period spanning late prenatal and early postnatal life, and that disruption of cerebellar function during this window can account for autism’s cognitive and social features. Limperopoulos et al. [206] provided complementary clinical evidence that perinatal cerebellar injury, particularly to the vermis, increases the risk of autism diagnosis by an order of magnitude. Direct in vivo evidence for prenatal/neonatal cerebellar involvement has been provided by Wagner et al. [214], who used neonatal functional magnetic resonance imaging, genetic, and behavioral follow-up data from the Developing Human Connectome Project (*n* = 198 term-born neonates, mean age 9.7 days) to demonstrate that elevated polygenic scores for autism predicted alterations in cerebello-cerebral functional connectivity within days of birth, with hemisphere-dependent differences in sensorimotor cerebellar connectivity with temporal cortex and hyperconnectivity between social–cognitive cerebellar regions and posterior cingulate cortex. Critically, these polygenic-risk-related connectivity patterns differed by biological sex and showed nominal associations with 18-month language ability, attention, and emotional reactivity. This neonatal evidence confirms that genetic liability for autism shapes cerebellar-cortical connectivity in the immediate postnatal period, consistent with the model’s prediction that the cascade is initiated by prenatal developmental events rather than emerging only with the appearance of behavioral symptoms. Two further infant studies converge with this account. Nair et al. [215] examined thalamocortical functional and structural connectivity in 6-week-old infants at high familial risk for autism (*n* = 52), reporting thalamic-prefrontal underconnectivity together with thalamic-occipital and thalamic-motor overconnectivity in high-risk infants, alongside atypical white matter integrity in thalamic-occipital tracts. Critically, these atypical connectivity indices at six weeks predicted atypical social development assessed between 9 and 36 months, including eye-tracking and diagnostic measures, providing a candidate early neurobiological marker that links prenatal-developmental connectivity differences to later behavioral outcomes. Marrus et al. [216] extended the developmental window into the toddler period using resting-state functional connectivity in a mixed cross-sectional and longitudinal cohort of infants at high and low familial risk for autism: at 12 months, motor and default mode network connectivity was correlated with walking, and at 24 months dorsal attention and posterior cingulo-opercular networks, with frontoparietal and subcortical networks additionally implicated for general gross motor function. These findings suggest that the cascade’s behavioral signature emerges in a dimensionally distributed range of motor function in early development. Pagani et al. [217] provided mechanistic specification at the synaptic level, demonstrating in Tsc2 haploinsufficient mice that mTOR-dependent increases in dendritic spine density produce autism-like stereotypies alongside cortico-striatal hyperconnectivity, and that pharmacological inhibition of mTOR fully rescues these phenotypes. Critically, the same cortico-striatal hyperconnectivity signature was demonstrated in children with idiopathic autism and was enriched for autism-dysregulated genes interacting with mTOR or Tsc2, with the transcriptomic signature expressed predominantly in a subset of children with autism. This finding causally links a specific synaptopathic mechanism to a measurable large-scale connectivity phenotype and provides a candidate biological substrate for one of several segregable autism subtypes. Zikopoulos, Garcia-Cabezas, and Barbas [218] further extended this framework by combining high-resolution comparative analysis of cortical gray and white matter architecture across humans and rhesus monkeys with examination of prefrontal pathways in autism, demonstrating significant changes in pathways between nearby prefrontal and distant areas in autism within a theoretical framework of systematic cortical variation. Evidence converges across neonatal functional connectivity [214], six-week-old thalamocortical connectivity [215], toddler motor-network connectivity [216], comparative primate prefrontal architecture [218], and synaptic-mechanism modeling [217]. That convergence positions the cascade not as a single sequential pathway but as a multi-level developmental phenomenon, with biological signatures detectable across several methodological windows. This evidence sits alongside the broader literature on cortical excitatory-inhibitory imbalance in autism [219], which Ma and Kwan [220] extended to the cerebellum itself. They reported that prenatal valproic acid exposure in mice produced decreased cerebellar weight and size, alongside reduced expression of glutamate decarboxylases 65 and 67, the GABA transporter GAT-1, and GABA transaminase. These molecular findings are reviewed comprehensively by D’Mello [221]. A prenatal environmental insult can therefore produce cerebellar hypoplasia together with altered excitatory-inhibitory balance within cerebellar circuitry. Human spectroscopic evidence points the same way: Johnson et al. [222], measuring glutamate and GABA in the amygdala-hippocampus region and the cerebellum across autistic children (*n* = 30), a clinical control group with sensory abnormalities but not ASD (*n* = 30), and typically developing children (*n* = 37), found elevated cerebellar glutamate in the autistic group, with the evidence for altered excitatory-inhibitory signaling clearer when analyses were restricted to males, and reported that lower cerebellar GABA/glutamate ratios were associated with more severe social impairment in both the autistic and the sensory-abnormality males. The authors read this as evidence that the cerebellum plays a transdiagnostic role in social impairment, which accords with the cross-condition position taken here and with the sex-modulation discussed below; these findings position prenatal cerebellar vermal hypoplasia as a plausible upstream contributor to the postnatal cortical connectivity phenotype rather than an incidental finding.

Several lines of evidence within this cascade are sex-modulated. Wagner et al. [214] reported that the neonatal cerebellar-cortical connectivity patterns associated with autism polygenic liability differed by biological sex, with hemisphere-dependent effects observed predominantly in male neonates. Pagani et al. [217] reported sex-specific behavioral effects of mTOR pharmacological rescue in their mouse model. The cascade framework does not require sex-specific mechanisms but is consistent with sex-modulated penetrance: if cerebellar growth regulation, Purkinje cell maturation, or activity-dependent cortical refinement are influenced by sex chromosome or sex hormone effects during the prenatal sensitive period, then identical genetic or environmental perturbations could yield different connectivity phenotypes by sex [223]. Ribeiro and Sherrard [224] propose one candidate molecular mechanism for this pattern, arguing that the transcription factor retinoid-related orphan receptor alpha (RORa), which is required for Purkinje cell development and is separately implicated in inflammation, circadian regulation, and sexual dimorphism, may link cerebellar developmental defects to the systemic and sex-differentiated features of neurodevelopmental disorders. This pattern is consistent with the well-documented sex difference in autism prevalence and warrants explicit attention in sample composition for connectivity research.

The developmental cascade framework also provides a principled account of differences between autistic disorder and Asperger syndrome, and, by extension, of the case-mixing problem in contemporary DSM-5 samples. Under DSM-IV, Asperger syndrome was distinguished from autistic disorder by typical early language milestones. Lincoln et al. [33], in seven case studies of individuals with Asperger syndrome, did not observe the consistent vermal lobule VI–VII hypoplasia that characterizes autistic disorder samples. The corpus callosum pattern also differed, with several cases showing anterior callosal enlargement rather than the posterior reduction typical of autistic disorder. This double dissociation, in which autistic disorder shows posterior vermal hypoplasia and posterior callosal reduction while Asperger syndrome shows anterior callosal enlargement without consistent vermal pathology, suggests that the two conditions may follow different developmental trajectories. The DSM-IV autistic disorder pattern is consistent with disruption originating in posterior cerebellar regions and propagating to posterior cerebral and association cortex, including circuits supporting language acquisition and integration. The Asperger pattern is more consistent with relatively preserved early posterior cerebellar-cortical development alongside altered frontal organization. Under DSM-5, both presentations are subsumed under the autism spectrum disorder diagnosis. Samples recruited under DSM-5 criteria therefore mix individuals whose underlying neuroanatomy may follow these biologically distinct trajectories, with predictable consequences for statistical power: group-level reductions in posterior vermis or posterior corpus callosum, which were robust in older samples enriched for autistic disorder, may attenuate in DSM-5 samples that include a substantial proportion of individuals with the Asperger-pattern neuroanatomy. This case-mixing effect represents a specific instance of the diagnostic-heterogeneity issue noted earlier and helps explain why some structural and connectivity findings well-established in older literature show weaker or more variable effects in current cohorts.

Direct-comparison and meta-analytic evidence from the voxel-based morphometry literature is consistent with the autistic-disorder versus Asperger-syndrome distinction at the broader cerebellar level, while remaining methodologically silent on the specific vermal lobule VI–VII hypothesis. As a primary direct-comparison anchor, McAlonan, Suckling, Wong, Cheung, Lienenkaemper, Cheung, and Chua [225] examined 17 children with high-functioning autism, 16 children with Asperger syndrome, and 55 typically developing controls, using whole-brain voxel-based morphometry in a single methodologically uniform sample. They reported distinct patterns of gray matter abnormality across the two clinical groups. That is not the quantitatively graded pattern that would be expected if Asperger syndrome simply represented a milder variant of autistic disorder. Aggregating this and other primary studies, Yu, Cheung, Chua, and McAlonan [226] conducted an anatomical likelihood estimation meta-analysis of 18 voxel-based morphometry studies, partitioning samples by majority diagnostic composition into a predominantly autistic disorder group (151 cases and 190 controls across nine studies) and a predominantly Asperger syndrome group (149 cases and 214 controls across nine studies). The summary maps differed in three theoretically informative respects. Both groups showed cerebellar gray matter reductions, but the autistic disorder group showed bilateral cerebellar reductions while the Asperger group showed only a right-sided cerebellar reduction. The Asperger map was sparser overall, with gray matter reductions concentrated in right-hemisphere structures, including the cerebellum, putamen, precuneus, and medial frontal gyrus, and gray matter excess concentrated in left-hemisphere parietal and ventral temporal regions, while the autistic disorder map showed bilateral excess across a wider set of cortical and subcortical regions. Yu and colleagues interpreted the broader pattern in the context of a distributed cortico-cerebellar network for multimodal language, consistent with the framework advanced here. Two methodological caveats are essential to the interpretation. The cerebellar peak Talairach coordinates reported in the autistic disorder and Asperger summary maps fall in lateral and inferior posterior cerebellar regions rather than at midline vermal coordinates. The method itself is also a constraint. Voxel-based morphometry with the smoothing kernels routinely used in this literature, 8 to 12 mm, combined with the standard adult spatial normalization applied in anatomical likelihood estimation meta-analyses, is not generally sensitive to vermal lobule VI–VII specifically. Adequate anatomical resolution at that scale requires manual region-of-interest tracing or cerebellar-specific atlases. Yu et al. [226] therefore provides convergent meta-analytic support, alongside the primary direct-comparison evidence of McAlonan et al. [225], for the autistic-disorder versus Asperger structural distinction at the level of cerebellar laterality and overall gray matter pattern, while leaving the central vermal lobule VI–VII claim of the framework advanced here awaiting direct region-of-interest-based meta-analytic synthesis.

#### 2.2.13. Cerebellar Internal Models, Auditory Prediction Error, and Early Language Acquisition

The developmental cascade outlined above describes a structural mechanism by which prenatal cerebellar vermal pathology disrupts the maturation of cortical connectivity in autism and, by extension, in related neurodevelopmental and genetic conditions and in the psychotic spectrum disorders (e.g., schizophrenia, schizoaffective disorder, and bipolar disorder) [140]. A complementary functional consequence concerns the cerebellum’s role as a generator of predictive internal models. Beginning with the work of Ito [83,227], and elaborated in contemporary syntheses by Sokolov, Miall, and Ivry [53], the cerebellum has been characterized as a substrate for the construction and refinement of forward models, that is, neural representations that anticipate the sensory consequences of actions, external events, and contextual regularities. These models support rapid prediction-error computation, with cerebellar output flowing through two pathways that differ markedly in temporal characteristics. The fast pathway projects from deep cerebellar nuclei via brainstem and midbrain routes to subcortical reward-related structures. It includes direct monosynaptic projections from the dentate nucleus to the ventral tegmental area [228], together with a disynaptic projection reaching the nucleus accumbens core and medial shell by way of the ventral tegmental area and the intralaminar thalamus, at an estimated conduction time of approximately 10 ms [229]. That timescale is compatible with the high rate of moment-to-moment sensory and social input characteristic of early development. Because the disynaptic pathway has been characterized in mouse optogenetic work and has not yet been replicated in primates, it is treated here as a strong candidate substrate rather than as an established feature of the human system. The slower pathway projects through cerebellar-thalamocortical loops to the cerebral cortex, where activity contributes to model refinement, conscious access to prediction mismatch, and longer-timescale behavioral adjustment. The distinction is consequential. The cerebellum’s primary contribution is to generate forward predictions rather than to register prediction error itself; downstream prediction-error registration depends on comparison of the cerebellar prediction with incoming sensory information at structures that receive cerebellar output. Because the term prediction error is used at more than one level in this literature, its senses are kept distinct here as they are in Lincoln [230]. Sensory prediction error, the mismatch between predicted and actual sensory input, is the quantity that cerebellar forward modeling most directly supports. Reward prediction error is the dopaminergic teaching signal carried by midbrain projections to the ventral striatum. Bayesian surprise, or the magnitude of belief updating, is the quantity indexed by the P3b component discussed below. These are not the same signal, and where the present account co-cites electrophysiological and computational measures, the intent is to display family resemblance across these levels rather than identity. In the developing infant, in whom incoming sensory and social signals arrive at rates that can exceed the temporal resolution of cerebellar-cortical loop activity, the fast subcortical pathway is therefore the principal site at which moment-to-moment prediction-error registration occurs, with cortical registration following on slower timescales through cerebellar-cortical tracts. The architecture is largely uniform across cerebellar regions, with motor lobules supporting sensorimotor predictions, posterior lobules (Crus I, Crus II, vermal lobules VI–VII) supporting cognitive, linguistic, and social predictions, and inferior vermal lobules contributing to autonomic and affective regulation [145,231,232]. Welniarz, Worbe, and Gallea [233] set out the forward model as a unifying account of cerebellar contributions to motor control and the sense of agency, without reference to autism. Kelly, Ochoa Escamilla, and Tsai [234] then applied that framework specifically to autism, proposing that cerebellar dysfunction compromises the implicit prediction-and-adaptation processes underlying social, linguistic, and adaptive behavior, and Kelly, Meng et al. [202] supplied the experimental circuit evidence by manipulating cerebellar-prefrontal projections to alter autism-relevant behaviors in mice. Kakei et al. [235] have recently extended this account by proposing that the Kalman filter, a computational model in which a system uses prior estimates and incoming observations to generate optimized predictions under uncertainty, captures the cerebellum’s contribution not only to motor control but also to cognitive, affective, and social predictive sequencing. They explicitly link cerebellar dysfunction in autism to impairments in mentalizing and narrative coherence, both of which depend on the temporal sequencing of causally related events. This formalization is consistent with the connection drawn in the present review between cerebellar predictive modeling and the uncertainty-reduction framework discussed below, and provides a candidate computational architecture for the cerebellum’s role in generating the predictive signals that, when compromised in autism, contribute to the broader phenotype.

On this view, the prenatal vermal lobule VI–VII hypoplasia documented in autistic disorder is doubly consequential. As described in the preceding section, it produces a structural cascade affecting cortical connectivity through disinhibited deep cerebellar nuclei and altered cerebro-cerebellar circuits. Functionally, it also constrains the cerebellum’s capacity to construct accurate, modality-specific predictive models during the same prenatal and early postnatal window in which experience-dependent learning of environmental regularities normally accelerates. The infant brain, with its concurrent cortical synaptic overproduction and cerebellar maturation, depends on the cerebellum providing well-tuned predictive signals to guide cortical refinement. Compromised cerebellar generation of predictive signals during this sensitive period [62] has cascading effects on both the fast subcortical reward pathway and the slower cortical pathway described above. It is therefore expected to interact with the cortical overgrowth pattern reported in early autism [111,112,114]. The cortex expands its synaptic repertoire on schedule, but without the predictive scaffolding required to prune and stabilize networks according to environmental contingencies. Development is biased toward locally robust but globally less integrated connectivity. The present account is compatible with but distinct from broader prediction-as-Bayesian-inference theories of autism [236,237,238,239], which describe altered weighting of priors and likelihoods at the cortical level without specifying a developmental substrate. The cascade-and-cerebellar-modeling framework specifies one such substrate: prenatal vermal hypoplasia limits the cerebellar generation of forward predictions, and the developmental cascade limits the cortical infrastructure that would otherwise integrate predictions with sensory input. These accounts are mutually constraining rather than mutually exclusive.

The auditory modality is particularly informative for testing this account, because typical early language acquisition depends critically on the infant’s ability to extract statistical regularities from the speech stream and to detect violations of those regularities. From the first months of life, typically developing infants demonstrate sensitivity to phonemic contrasts in their native language, prosodic patterns, transitional probabilities across syllables, and the temporal contingencies that link adult vocalizations to infant social signals [240,241]. The detection of mismatches between predicted and observed auditory input drives the gradual narrowing of phonemic categories, the segmentation of continuous speech into word-like units, and the binding of phoneme sequences to meaning through contingent social interaction. Contingent caregiver responses, including the contingent smile elicited by an infant’s vocalization or eye contact, function as predictable consequences whose timing and form scaffold the infant’s emerging models of social communication [242,243]. A cerebellar substrate that generates degraded predictive signals during this period would be expected to disrupt this entire cascade: phoneme-to-meaning linking, segmentation of word forms, and the formation of expectations about contingent social responsiveness would all be impaired in proportion to the degree of cerebellar dysfunction. The clinical observation that infants who later receive an autism diagnosis show reduced contingent social smiling, atypical response to name, delayed word recognition, and altered auditory event-related potentials to speech stimuli [240,244] is consistent with this account. The dopaminergic-reinforcement layer of this account has begun to be empirically anchored in infants. In adults, direct gaze from an unfamiliar face activates the ventral striatum, a core dopaminergic reward structure, with activation tracking perceived attractiveness [245], establishing the adult-brain foundation for a gaze-as-reward signal. In 7-month-old infants, Tummeltshammer, Feldman, and Amso [246] used spontaneous eye-blink rate and pupil dilation, both validated as peripheral indices of striatal dopaminergic activity [247], to probe reward learning, reporting that both indices showed graded responses to social-emotional stimuli that increased in reward value (male stranger to female stranger to mother), with the largest responses to the mother. These findings indicate that dopaminergic reward circuitry is functionally engaged by social stimuli in mid-infancy, and the cerebellum-to-ventral-tegmental-area and cerebellum-to-accumbens pathways documented by Carta et al. [228] and D’Ambra et al. [229], reviewed alongside the wider literature on cerebellar contributions to reward by Manto et al. [248], provide a candidate anatomical substrate by which cerebellar generation of accurate predictions about contingent social and vocal consequences could drive positive prediction-error signaling at the ventral tegmental area and downstream reinforcement of contingent social engagement [230]. On this account, compromised cerebellar prediction in early development should attenuate this reinforcement loop, predicting the reduced contingent social smiling, gaze-maintenance, and response to name documented in prediagnostic infants. Recent infant connectivity work has begun to provide more direct evidence: Okada et al. [249] reported that atypical cerebellar functional connectivity at 9 months of age predicted delayed socio-communicative profiles in infants at high and low familial risk for autism, suggesting that early cerebro-cerebellar circuit alterations precede and forecast the language and social delays that define the diagnosis. Dean, Freeman, and Lainhart [250] reviewed the broader infant-sibling literature, noting that subcortical and cerebellar volumes differ at 4 to 6 months of age and that atypical cortical responses to social stimuli are detectable across this same window, alongside increased extra-axial cerebrospinal fluid at 6 months and cortical hyper-reactivity at 8 months. Yi et al. [251] extended this picture into the preschool period (*n* = 31 autism, *n* = 30 controls), using multimodal magnetic resonance imaging to identify consensus abnormal connections in cortical–cerebellar and subcortical–cerebellar circuits, with the thalamus and basal ganglia particularly implicated. The convergence of cerebellar-connectivity differences across neonatal, six-week, four-to-six-month, nine-month, preschool, and toddler windows, in independent samples and using distinct imaging modalities, supports the framework’s specification that the cerebellar substrate disruption is present from early in development rather than emerging with the appearance of behavioral symptoms.

This account makes a distinction worth examining empirically with developmental dysphasia (or specific language impairment). Children with developmental dysphasia also present with delayed expressive and receptive language, but the auditory processing literature originating with Tallal and Piercy [252,253,254] has documented a different proximal mechanism: a difficulty processing rapidly successive acoustic events, fast rise-time transitions, and short intersyllabic intervals, with consequent impairment in the discrimination of stop consonants and other rapidly changing speech cues. This is fundamentally a temporal-resolution deficit at the level of auditory analysis, not a generalized failure of predictive modeling, and it occurs without the cerebellar vermal pathology characteristic of autistic disorder. Both conditions can therefore produce delayed and atypical language, but through distinct routes: developmental dysphasia by limiting the fidelity of the acoustic input on which predictions are built, autistic disorder by limiting the predictive architecture itself. Children with developmental dysphasia generally retain typical social motivation and contingent responsiveness, consistent with intact non-auditory predictive functions, whereas children with autistic disorder show broader prediction-related impairments extending well beyond the auditory modality, consistent with a more general compromise of cerebellar predictive modeling [238,255]. The autism-versus-developmental-language-disorder contrast was directly examined by Lincoln, Dickstein, Courchesne, Elmasian, and Tallal [85], who compared intellectually typical adolescents and young adults with autistic disorder, individuals with receptive developmental language disorder, and matched controls on auditory processing tasks derived from the rapid-temporal-processing paradigm developed in Tallal’s laboratory. Subsequent event-related potential work from the same research program documented a graded dissociation in higher-order auditory processing across the two clinical groups. Courchesne, Lincoln, Yeung-Courchesne, Elmasian, and Grillon [186] reported reduced P3b and Nc amplitudes specific to autistic disorder, indicating attenuated endogenous attention and memory-related responses to important information. Lincoln, Courchesne, Harms, and Allen [84] extended this finding using a contextual probability paradigm, demonstrating that when children with autistic disorder, children with receptive developmental language disorder, and typically developing controls were presented with frequent and infrequent randomly intermixed auditory stimuli, only the autistic disorder group showed an abnormally small P3b amplitude. The authors interpreted this as evidence of a specific difficulty among children with autistic disorder in modifying their expectancies in response to contextually relevant sequences of auditory information. Lincoln, Courchesne, Harms, and Allen [256] subsequently examined earlier auditory ERP components (N1, P2) thought to reflect primary and secondary auditory cortical processing and found partial abnormalities in both clinical groups in indexing changes in stimulus intensity. Across this research program, the pattern that emerged was one of shared deficits at the level of basic auditory cortical responses to stimulus parameters, but autism-specific deficits in higher-order predictive and contextual processing of auditory information. This dissociation, established more than three decades ago, directly anticipated the contemporary framework of distinct predictive-modeling versus input-fidelity routes to language impairment in autism and developmental language disorder, and provides convergent ERP-level support for the broader framework outlined here. Functional MRI evidence at an earlier age converges with this electrophysiological dissociation. Redcay and Courchesne [257] scanned 2- to 3-year-old children with autism spectrum disorder during natural sleep using a forward-and-backward speech paradigm and compared them with both chronological-age-matched and mental-age-matched typically developing controls. Three findings are theoretically pivotal. First, in comparison to mental-age-matched controls, the autism group showed reduced activation across an extended network during forward speech, including frontal, temporal, parietal, occipital, and cerebellar regions, consistent with the cascade model’s prediction that the typical 2- to 3-year-old language-acquisition network is incompletely engaged in autism. Second, in comparison to chronological-age-matched controls, the autism group recruited right and medial frontal regions more strongly, while controls preferentially recruited left frontal and temporal regions, and a within-group hemispheric comparison confirmed a trend toward greater right-hemisphere recruitment in autism and greater left-hemisphere recruitment in typical controls. Third, dimensional analyses within the autism group revealed that activity in the right superior temporal sulcus, middle temporal gyrus, and inferior frontal gyrus during forward speech was positively correlated with receptive language age and negatively correlated with autism severity on the Childhood Autism Rating Scale, indicating that early right-hemisphere recruitment indexes a partially compensatory developmental trajectory rather than a static endpoint. Because data were acquired during natural sleep, the deviant lateralization cannot be attributed to cognitive strategy, anxiety, or attentional state, confounds that limit the interpretability of awake fMRI in older high-functioning samples. These findings extend the ERP dissociation reviewed above into the whole-brain fMRI domain at the age of disorder emergence, document reduced cerebellar engagement during speech processing concurrent with deviant cortical lateralization, and align with the cascade-and-predictive-modeling framework outlined here, in which compromised cerebellar predictive scaffolding interacts with cortical synaptic overproduction during the first three postnatal years to bias language-system specialization away from typical left-hemisphere dominance. Direct empirical support for the distinctness of these two routes has been provided by Swanson et al. [86] in a prospective longitudinal study of infants at high familial risk for autism. Comparing infants later diagnosed with autism (*n* = 86), high-risk infants with signs of early language delay but no autism (*n* = 41), and high- and low-risk unaffected infants, Swanson and colleagues found that although the autism and language-delay groups showed comparable delays in expressive and receptive language at 12 and 24 months of age, the two groups diverged in two informative ways. First, only the autism group displayed atypical receptive-expressive language profiles at 24 months, with balanced or expressive-advantage patterns, whereas the language-delay group, like typically developing controls, showed a normative receptive advantage. Second, and more striking, the associations between 12-month volumes of thalamus, amygdala, and caudate nucleus and 24-month language outcomes were disordinal across the two groups: in the language-delay infants, smaller subcortical volumes were associated with more normative language profiles, while in the autism infants the relationships were flat or in the opposite direction. Follow-up analyses indicated that autism infants who also met criteria for language delay showed brain-behavior associations indistinguishable from autism infants without language delay, and both autism subgroups differed from the language-delay-only group. A comparable decoupling has been reported at preschool age with the cerebellum explicitly among the regions examined. Xin et al. [258], imaging 24 children with ASD and 20 without between 12 and 52 months, found greater global gray matter volume in the ASD group but no regional gray matter differences, and reported that in the children without ASD gray matter volume in bilateral prefrontal cortex and cerebellum correlated significantly with language scores while no such correlation was present in the children with ASD. The pattern is the same phenomenon Swanson et al. [86] documented at 12 to 24 months with subcortical structures, observed independently at a later age and in the two regions this framework identifies as central. It should be weighted cautiously, because the absence of a correlation in a sample of 24 is weak evidence for the absence of an association, and the study found no regional volumetric group differences at all. With that caveat, the convergence is notable: what distinguishes the autism groups in both studies is not the volume of the structures but the breakdown of the normal coupling between those structures and language outcome, which is what an account positing a compromised predictive-modeling substrate rather than a simple volumetric deficit would predict. These results provide direct evidence that comorbid language delay in autism reflects a fundamentally different neurobiological substrate than language delay occurring outside the autism spectrum, consistent with the framework outlined here in which autism affects the predictive-modeling architecture itself while developmental language disorders constrain the inputs to otherwise intact predictive systems.

At a higher level of abstraction, the same framework helps account for the differential difficulty individuals with autistic disorder display with contextual modeling, which encompasses the inference of probabilistic regularities across time, settings, and social partners that underlies pragmatic language use, theory of mind, and adaptive social behavior. If posterior vermal and Crus I/II circuits provide the substrate for constructing and updating these context models, then their early disruption should produce difficulties in modulating predictions by context that extend beyond any specific sensory modality. This prediction is consistent with the broader literature on context insensitivity in autism, including weak central coherence findings, reduced use of prior probabilities in perceptual decisions [237], and difficulty with implicit social-pragmatic inference. Importantly, the framework predicts that this contextual modeling difficulty should be most pronounced in autistic disorder phenotypes characterized by early language delay, where vermal lobule VI–VII hypoplasia is most consistently observed. In Asperger syndrome, where Lincoln et al. [33] found preserved vermal volume and a distinct callosal pattern, the predictive modeling architecture is comparatively intact. The cognitive and social differences in Asperger syndrome would, on this view, arise primarily from cortical-level alterations in the integration and weighting of model outputs rather than from a fundamental compromise of the predictive substrate itself. This dissociation provides an additional theoretical motivation, beyond the structural double dissociation already noted, for the case-mixing concern: combining individuals whose connectivity differences originate in cerebellar predictive-model failure with individuals whose differences originate in cortical integration of otherwise intact predictions is likely to obscure both patterns at the group level.

#### 2.2.14. Why Language Is the Earliest Casualty

The specificity of the language phenotype requires explanation, since the framework posits a system-general disruption of cerebellar predictive scaffolding rather than a language-specific lesion. The explanation is not one of processing rate. Tallal and Piercy [252,253,254] established that developmental dysphasia involves impaired rate of non-verbal auditory processing, and that deficit is characteristic of developmental receptive language disorder rather than of autistic disorder. The electrophysiological dissociation reviewed above bears this out directly. Lincoln, Courchesne, Harms, and Allen [256] found N1 and P2 abnormalities, indexing primary and secondary auditory cortical processing, in both clinical groups, whereas reduced P3b amplitude was specific to autistic disorder [84,186]. Lower-level auditory processing is therefore not what distinguishes the autistic disorder phenotype. The failure is at the level of predictive template mapping: the construction and updating of expectancies against which incoming sequences are evaluated.

This locates the deficit anatomically as well as functionally. The computational resources for template construction reside in the hemispheric extensions of lobules VI and VII, which is where the modern literature localizes cerebellar contributions to language and to social prediction [25,232,259]. Crus I and Crus II are the hemispheric extensions of lobule VII, so the right lateral localization reported in that literature and the vermal localization reported in the autism structural literature describe the same transverse lobular zone rather than competing sites. The vermal portion of that zone contributes to coordination across hemispheric computation rather than performing it. On this account, midline vermal deviation degrades the coordination of a distributed predictive operation whose substrate is largely lateral, which is why a midline structural finding can produce a phenotype whose functional signature appears in lateral cerebellar systems.

Language is the earliest and most visible casualty of that degradation because language acquisition in the first two years depends heavily on predictive template mapping over rapidly arriving sequential input [241]. The consequence is not a language-specific disorder but a general failure of contextual modeling whose earliest expression is linguistic, and whose later expression is the pragmatic and social contextual difficulty that defines the phenotype in older individuals.

#### 2.2.15. Pragmatic Competence, Early Social Attention, and Evidence from Intervention

Pragmatic communication is often described in terms of its failures rather than its components, which obscures what the underlying operation requires. Its central features are prosodic modulation of tone and volume, joint referencing, and pointing. Each of these demands continuous adjustment of output against a running model of the listener, the setting, and what has already passed between the participants, and each demands it at a rate that excludes deliberate evaluation. Prosody is the clearest case, and the one where the motor analogy is not an analogy at all: tone and volume are produced by the vocal apparatus, so their contextual modulation is a forward model operating through a motor effector on a social variable. Joint referencing and pointing are the developmentally earliest expressions of the same operation, emerging within the first year, well before the language delay that defined the cohorts in which the cerebellar findings were established. If the framework advanced here is correct, these should fail earlier than language does, both being expressions of a single predictive deficit sampled at different developmental points.

The infant-directed speech literature provides direct evidence on the prosodic component. Motherese is exaggerated affective prosody, which makes it the pragmatic signal presented as stimulus rather than as response. Pierce et al. [260], studying 653 toddlers aged 12 to 48 months, found that fixation on motherese was high in toddlers without autism spectrum disorder and significantly reduced in those with it, with the reduction associated with weaker language and social ability. Xiao et al. [261], combining natural sleep functional magnetic resonance imaging with eye tracking, identified four distinct clusters of toddlers, in which the autism cluster showing the weakest superior temporal responses to affective speech also showed the poorest social and language abilities and the lowest behavioral preference for motherese. Two features of this work bear on the present account. The measure operates at 12 months, before the language phenotype is expressible, which is consistent with the developmental ordering the framework predicts. And the authors describe reduced attention to motherese as marking a subtype within autism spectrum disorder rather than a property of the diagnosis as a whole, which is an independent arrival at the stratification argument developed earlier in this review.

The interpretation offered by those authors is cortical, attributing the effect to impaired development of temporal cortical systems that would ordinarily respond automatically to parental emotional speech. The present framework does not dispute that localization but places it downstream. Degraded cerebellar predictive scaffolding of superior temporal circuits during the period when those circuits are being tuned would produce precisely the reported pattern, and the framework therefore reinterprets rather than contradicts the finding. The distinction is testable, since a cerebellar-origin account predicts that cerebellar measures should covary with motherese preference within autism cohorts whereas a primary cortical account does not require this.

Evidence from early intervention bears on the framework in a way that initially appears to contradict it. Dawson et al. [262] randomized 48 children diagnosed between 18 and 30 months to the Early Start Denver Model or to community referral for two years, reporting significant improvements in intelligence quotient, adaptive behavior, and diagnostic status in the intervention group. Dawson et al. [263], reporting electroencephalographic outcomes from the same trial, found normalized patterns of brain activity in the intervention group, including differences in the peak latency of the Nc response to faces relative to objects. That component is one of the two whose attenuation Courchesne, Lincoln, Yeung-Courchesne, Elmasian, and Grillon [186] reported as specific to autistic disorder, so the electrophysiological signature that anchored the original dissociation is the same signature that responded to intervention.

The apparent contradiction is worth stating plainly rather than leaving it implicit. If the phenotype originates in a prenatal structural deviation of cerebellar vermal lobules VI and VII, why should two years of behavioral intervention beginning at 18 months normalize the electrophysiology? The framework’s answer is that no claim is made that the structural deviation changes. What the cascade claims is that degraded cerebellar predictive scaffolding biases cortical network formation during a window in which synaptic density peaks and activity-dependent pruning remains active, a window still open at 18 to 30 months. Intensive, naturalistic, high-frequency social exchange of the kind the Early Start Denver Model delivers supplies externally the contingency structure that intact predictive scaffolding would otherwise supply internally. On this reading, early intervention does not repair the cerebellar substrate. It substitutes environmental regularity for a degraded internal signal during the period when cortical connectivity is still being established.

This reading generates a specific and falsifiable prediction. Intervention effects should be graded by position within the developmental window, with larger effects for earlier initiation and diminishing returns as cortical pruning completes, and the gradient should be steeper than would be expected from duration of treatment alone. A further prediction follows from the stratification argument: if the cerebellar account is specific to the language-delayed phenotype, intervention response should differ between strata defined by early language status rather than being uniform across the contemporary spectrum.

#### 2.2.16. Convergence with Cerebellar Predictive-Processing Accounts

The account developed here arrives at a position that several groups have reached independently, and the convergence is worth making explicit rather than leaving for a reader to notice. Van Overwalle and colleagues have argued across a decade of work that the posterior cerebellum learns and predicts social action sequences, and that this sequencing function underlies cerebellar contributions to mentalizing [264,265]. Their consensus paper assembled twenty-one authors around that position and included a treatment of cerebellar contributions to social behavior in autism. Stoodley and Tsai [266] developed a closely related account under the heading of adaptive prediction for social contexts, and Frosch, Mittal, and D’Mello [267] set out a predictive-processing framework for cerebellar contributions to social cognition in autism specifically. The proposal that cerebellar forward modeling extends from motor sequences to social and contextual content is therefore not novel to this review, and the present framework should be read as converging with that literature rather than competing with it.

The proposal that prediction is a fundamental cerebellar function, and that its failure is central to autism, has a direct antecedent in this literature. Courchesne and Allen [71] argued that prediction and preparation are among the cerebellum’s basic operations, extending beyond motor control to the anticipation of sensory, cognitive, and social events, and applied that account to autism specifically. The framework developed in the present review is a descendant of that proposal rather than an alternative to it. What has been added since is threefold: the anatomical specificity supplied by the bidirectional vermal findings, the developmental cascade linking the prenatal perturbation to the cortical connectivity signature, and the identification of the diagnostic stratification required for the effect to be detectable in contemporary samples.

A further extension bears on why predictive failure should matter motivationally rather than only computationally. Lincoln [230] argues that the reduction in predictive uncertainty operates with the functional and physiological signatures of a primary reinforcer, mediated by prediction error signaling in nucleus accumbens and lateral habenula circuitry and gated by the fast cerebellar projection to that circuitry. On that account, the cerebellum does not merely supply predictions for cortical use. It participates in the reinforcement system that makes successful prediction intrinsically motivating, and therefore in the process by which an infant is driven to seek out the contingent social and linguistic input from which predictive templates are built. Degraded cerebellar prediction would then have two consequences rather than one: the templates are built less well, and the reinforcement that ordinarily drives the infant toward the experiences required to build them is itself attenuated. This gives the cascade a motivational limb alongside its computational one, and it offers a mechanism for the reduced orientation to socially contingent input documented in the infant literature reviewed earlier.

Three things distinguish the account advanced here, and stating them locates the contribution precisely. The first is the structural anchor. The predictive-processing accounts largely proceed from functional and connectivity evidence and do not require a specific structural lesion; the present framework ties the predictive deficit to a documented anatomical finding at vermal lobules VI and VII and their hemispheric extensions, which makes it more falsifiable and more vulnerable. The second is the developmental cascade. Those accounts describe what the cerebellum does and what happens when it does it poorly; the cascade specifies the intervening steps, from prenatal vermal deviation through altered excitatory drive in cerebello-thalamo-cortical pathways during a defined sensitive period to the cortical connectivity signature, and thereby commits to a mechanism that could be shown to be wrong. The third is the stratification argument. The predictive-processing literature has generally taken the autism spectrum as given; the present review argues that the cerebellar findings are specific to the language-delayed phenotype and that failure to stratify is a principal source of the inconsistency in the structural literature.

The D’Mello, Moore, Crocetti, Mostofsky, and Stoodley [8] result bridges these positions. It comes from the same laboratory group that developed the social predictive-processing account, and it reports the stratified finding that the present framework predicts. Read together with the sequencing evidence, it suggests that the two studies describe the same phenomenon at different levels: a cerebellar predictive operation whose developmental failure is most severe in the subgroup defined by early language delay, and whose functional signature in adulthood is the impaired social sequencing that the Van Overwalle group has characterized. On that reading, the social prediction findings and the structural findings are not competing localizations of autism, but successive views of one developmental process, and the cerebellar contribution to social cognition described in the recent literature is the mature expression of a scaffolding failure that occurred years earlier.

In companion work, Lincoln [230] has proposed that the reduction in predictive uncertainty is a candidate primary reinforcer, mediated by positive prediction-error coding within nucleus accumbens and lateral habenula (NAc-LHb) opponent-process circuitry and gated and refined by later cerebellar and cortical additions, with successful predictive resolution generating intrinsic reward that ordinarily sustains engagement with the contingent social and linguistic environment. That account is explicit that the claim has the status of a candidate rather than an established primary reinforcer, and it specifies the experiment capable of deciding it. An arbitrary neutral cue is paired with the resolution of a probabilistic prediction, under novelty, arousal, task-completion, and effort controls, and with an explicit establishing-operation analog. If the unconditioned reinforcer hypothesis is correct, acquisition and extinction dynamics should be quantitatively comparable to those produced by pairing with food or water. If uncertainty reduction is instead a generalized conditioned reinforcer, they should diverge. The two frameworks are mechanistically continuous rather than only thematically compatible, because the fast disynaptic cerebellum-to-accumbens projection described above is the specific route by which that account links cerebellar forward modeling to subcortical reinforcement, and the neocerebellar vermis is the same structure in both. The cascade-and-predictive-modeling framework developed here does not depend on that motivational proposal, but is consistent with it: reduced cerebellar predictive modeling would attenuate the reinforcement value of contingencies depending on accurate prediction, providing one mechanism through which the cascade’s effects on behavior could become self-reinforcing across development. A comparative treatment of this literature, which this review identifies as a necessary next step, reviews the broader comparative connectivity literature across autism and other clinical populations, including the transdiagnostic cerebellar findings that converge with the substrate framework developed here. The combination remains testable: the cascade and predictive-modeling components yield conjoint, specific predictions for infant studies combining cerebellar functional connectivity, electrophysiological measures of auditory and contextual prediction error, and behavioral indices of socially contingent learning.

A further consequence of the same architecture bears on restricted and repetitive behavior. This subsection and the one that follows are offered as an exploratory extension of the framework, resting on a thinner evidentiary base than the cerebellar developmental argument that precedes them, and are labeled as such so that the status of the two is not confused. It follows from the temporal asymmetry described above. No additional assumption is required. Successful predictive resolution is reinforcing, and the fast disynaptic route delivers that reinforcement at approximately 10 ms, whereas cortical evaluation requires several hundred milliseconds. Under degraded cerebellar prediction, the behaviors most reliably reinforced will therefore be those whose consequences a degraded forward model can still anticipate. Externally generated input is what such a model predicts poorly, because it arrives rapidly, varies across instances, and is contingent on agents whose behavior the model does not capture. Self-generated invariant action is what it predicts well. A stereotyped movement, a repeated vocalization or echoed phrase, and a rehearsed sequence of thought each produce proprioceptive, auditory, and visual consequences that are close to identical on every occasion, and each therefore yields reliable prediction resolution on the fast subcortical route. The proposal advanced here is that behavior of this kind may be reinforced not despite its failure to modify the environment but in part because of that failure, since invariance is what makes the consequence predictable and predictability is what makes the resolution reliable. On this reading, the category of automatically reinforced behavior in the functional analytic literature [268], in which maintaining consequences are internal and not socially mediated, acquires a candidate mechanism at the level of circuitry, with the operative consequence identified as prediction resolution rather than sensory product alone. The timing relations then close the loop. Reinforcement on the fast route is collected within milliseconds, whereas the context updating indexed by the P3b at 300 to 600 ms and the semantic prediction error indexed by the N400 at approximately 400 ms [269] are followed in turn by the working memory operations required to represent an alternative and select it. A high-rate repetitive behavior can therefore generate an inter-reinforcement interval shorter than the interval cortical evaluation requires, so that the next resolution arrives before the preceding one has been evaluated. Cortical regulation on this account is not overridden so much as outpaced. The attenuation of P3b amplitude reported as specific to autistic disorder [84,186] is consequential here in a way it is not elsewhere in this review, because it implies that the slower of the two competing processes is also the weaker one. The predicted behavioral signature is the one commonly observed clinically: acquisition and maintenance of repetitive behavior that are rapid and durable, contingency-based revision that is slow, elevation of repetitive behavior under conditions of novelty, demand, or environmental unpredictability, and reduced sensitivity to changing reinforcement contingencies of the kind reported in adults with autism [270]. It is consistent with the impairment in rapid shifting of attention associated with neocerebellar vermal deviation [271], and with the animal work reviewed earlier in which cerebellar perturbation produces repetitive-behavior phenotypes alongside altered cortico-striatal connectivity [75,213,217]. The account is offered as a hypothesis rather than a demonstration, and it is testable in at least two ways. Within autistic disorder samples, P3b amplitude and latency should covary with the rate and the invariance of repetitive behavior after measured intelligence is accounted for. And experimentally increasing the predictability of the ambient environment should reduce the reinforcing efficacy of self-generated invariance, and with it the rate of the behavior, whereas an account in which repetitive behavior is maintained by sensory product alone does not require this.

The second consequence runs in the opposite direction and is the more consequential of the two. If invariant self-generated action is what a degraded forward model can predict, then socially contingent exchange is precisely what it cannot. Pragmatic exchange is the hardest prediction problem an infant faces, because the signal is rapid, multimodal, probabilistic, and generated by another agent whose behavior is not fully modelable. On the account developed here, the two consequences are not separate phenomena but a single reallocation, and the matching law supplies its quantitative form: behavior is allocated in proportion to obtained reinforcement, so as the certainty yield of self-generated invariance rises relative to that of socially contingent exchange, allocation shifts toward the former and away from the latter. What is lost is not a behavior but a developmental input. The model predicts that joint attention, referencing, prosodic modulation, and turn-taking are the earliest tier to fail, and that they fail before language because they are the operations through which the contingency structure of social exchange is first sampled. Language acquisition sits immediately above that tier and depends on sequential prediction over rapidly arriving input, which is why early language onset delay follows, and why it was serviceable as the DSM-III and DSM-IV criterion that defined the cohorts in which the vermal findings were established. Above language sits everything that language makes possible: propositional representation, derived relational responding, recursive evaluation, and the context-sensitive higher-order mental operations whose failure this review has described as context-blindness. Each tier is constructed on the one beneath it, so a single degradation of predictive scaffolding does not produce three deficits, but one deficit expressed three times at ascending levels of abstraction, with severity compounding upward because each tier inherits the impoverished input of the tier below. This is the cascade in its behavioral form, and it is what makes the framework a developmental account rather than a description of a static phenotype. The two consequences are therefore complementary rather than independent: reinforcement withdrawn from the pragmatic tier is the same reinforcement that repetitive behavior reliably supplies, and an intervention that raises the predictability of social exchange should move both, reducing the rate of repetitive behavior and increasing engagement with contingent input, which is one reading of why the Early Start Denver Model results reported earlier take the form they do. Figure 1 presents the framework as a whole, with the two limbs of the account and the ascending tiers of the behavioral cascade shown together.

### 2.3. Methodological Sources of Variability in Connectivity Findings

Beyond inherent biological heterogeneity in autism, methodological choices substantially influence whether studies report overconnectivity, underconnectivity, or no group differences, and the resulting variability complicates direct comparison across the connectivity literature. Hull et al. [272], in a systematic review of resting-state fMRI studies of autism, observed that reported findings varied with the analytic approach used. Seed-based analyses tended to identify region-specific connectivity differences; independent component analysis (ICA) more frequently identified distributed network differences; graph-theoretic approaches highlighted topological properties not captured by either of the former methods; and voxel-wise whole-brain analyses produced different findings than region-of-interest analyses derived from a priori network definitions. Picci, Gotts, and Scherf [273] similarly argued that the field had become locked into a generalized under/over-connectivity dichotomy partly because methodological variation across studies obscured the conditions under which specific patterns emerged.

Several specific factors contribute to cross-study variability. Acquisition factors, including scanner field strength, pulse sequence, voxel size, repetition time, and slice prescription, affect signal-to-noise ratio and the spatial scale at which connectivity is measured. Preprocessing choices matter as well. Differences in motion correction, registration, slice-timing correction, nuisance regression, and global signal regression produce systematic differences in connectivity estimates even when applied to the same raw data [274]. Head motion is a particularly consequential confound, because children with autism, especially younger and lower-functioning individuals, tend to move more in the scanner, and head motion produces spurious connectivity effects that mimic reduced long-range and elevated short-range connectivity [101,275,276]. Earlier studies that did not stringently control for motion may have reported connectivity differences partly attributable to motion artifact. More recent studies using motion scrubbing and stringent quality control have reported smaller effect sizes.

#### Subgroup Heterogeneity and the Interpretation of Group Means

A general methodological principle underlies much of what follows, and it is worth stating in the abstract before applying it. A comparison of group means is informative only when the clinical group is homogeneous with respect to the measure being compared. Where a sample contains subgroups that deviate from the typical value in opposite directions, the group means will approach the control mean regardless of how large the individual deviations are, and a null result will be obtained from data in which every participant differs from typical. Where a sample contains one subgroup in which an effect is present and another in which it is absent, the group means will be attenuated in proportion to the mixture. In neither case does the null describe the biology. It describes the composition of the sample.

This principle was first applied to the cerebellar literature in autism more than three decades ago. The initial reports of reduced posterior vermal size [20,22] were followed within four years by several studies reporting no significant difference in posterior fossa measures between autistic and comparison samples [277,278,279,280], and later by a report of increased cerebellar volume [281]. Courchesne, Townsend, and Saitoh [70] proposed that samples composed of both hypoplastic and hyperplastic subgroups would yield a mean near the typical value precisely because the two opposite deviations cancel, so that a null group difference would be expected even where cerebellar abnormality was present throughout. A companion paper addressed the measurement conventions themselves [282], and Filipek [283] reviewed the exchange and concluded that the methodological questions were not yet settled.

The identification of the bidirectional distribution described earlier, with approximately 86 percent of an autistic disorder sample showing hypoplasia and approximately 12 percent showing hyperplasia at the same vermal lobules [21], supplies the empirical basis for that interpretation. It is not offered here as an adjudication of the earlier disagreement, which turned on measurement questions that this review is not positioned to resolve. It is offered as the observation that makes the interpretation available: once a clinical group is known to contain subgroups deviating in opposite directions at the same structure, the group mean ceases to be the appropriate test statistic for that structure.

Sample composition in this literature also differed along dimensions independent of measurement technique. Studies varied in whether diagnosis was established by consensus between independently conducted clinical evaluations, in whether standardized instruments such as the Autism Diagnostic Interview, Revised and the Autism Diagnostic Observation Schedule were required, in whether neurological and genetic evaluation was performed to exclude conditions such as fragile X syndrome, and in whether participants with intellectual disability were included or excluded. They varied as well in acquisition parameters, including slice thickness, slice angle, and the anatomical conventions used to define lobular boundaries. These are differences in what was sampled and what was measured, not in the quality of the investigators. They are noted because a group of studies that differ this substantially in ascertainment should not be expected to converge on a single effect size, and their divergence carries less evidential weight against the original finding than a simple count of positive and negative results would suggest.

The relevance of this history to the present argument is structural rather than historical. The explanation advanced later in this review for the contemporary attenuation of cerebellar findings, namely that samples ascertained under broadened diagnostic criteria mix a subgroup in which the effect is present with a subgroup in which it is not, is the same argument applied to a different source of heterogeneity. In 1994, the heterogeneity was directional, within a diagnostically narrow sample. In the contemporary literature, it is diagnostic, across a sample that is directionally uninformative because it was never stratified. The mechanism is identical in both cases: a group mean computed over a mixture describes the mixture rather than its components. The framework developed here is therefore not a new explanation for non-replication but an extension of an existing one to a new source of sample heterogeneity.

One further implication follows for the moderation result discussed above. Stanfield et al. [32] reported larger cerebellar effects in samples of lower measured intelligence, which is consistent with the case-mixing interpretation but also with a rival account in which the cerebellar finding tracks intellectual impairment rather than the autistic disorder phenotype. The rival account is weakened by the observation that several of the foundational samples excluded participants with intellectual disability by design, so that the vermal findings in those cohorts cannot be attributed to intellectual impairment. To the extent that measured intelligence varied systematically with ascertainment rigor across this literature, IQ moderation at the level of pooled studies may index differences in sample composition rather than a moderating effect of intelligence as such. The discriminating test is direct: within a contemporary sample matched on measured intelligence, the framework predicts that vermal lobule VI and VII measures will still separate individuals with retrospective early language delay from those without it. A null result under that design would count against the framework.

Sample composition also matters. Connectivity in autism follows non-linear age-dependent trajectories with early overconnectivity transitioning to later underconnectivity [284], so samples differing in age range yield different findings even when sampling from comparable diagnostic criteria. Most older studies enrolled predominantly male participants, raising questions about generalizability across sex. Diagnostic heterogeneity further compounds these issues, as discussed in the preceding section. King et al. [25] reported that connectivity differences between autism and typical development showed limited replication across the 17 ABIDE I sites once site and motion were stringently controlled, suggesting that effect sizes for many findings are smaller than initially reported and that some site-specific effects may have been mistaken for diagnostic effects. The cross-site replication concern bears directly on the cascade model: if the cortical-connectivity phenotype were a robust and direct downstream consequence of a circumscribed prenatal cerebellar perturbation, larger and more reliable effects should be expected. The observed modest and variable effects therefore require either (a) substantial biological heterogeneity within ASD, consistent with the DSM-IV/DSM-5 case-mixing concern and the hyperplasia/hypoplasia subgroup distinction, (b) measurement noise that obscures genuine effects, or (c) both. Large multisite mega-analyses have begun to clarify which findings are robust across sites and acquisition pipelines [285], and emerging biologically defined subgrouping methods [217] may help resolve which findings reflect a single mechanistic route versus several converging mechanisms. These methodological sources of variability help explain why the connectivity literature in autism contains apparent contradictions, why effect sizes have generally been modest, and why older small-sample single-site findings should be interpreted with appropriate caution.

## 3. Future Directions and Boundary Conditions

Cerebral connectivity differences in autistic disorder, as that term was defined under DSM-III and DSM-IV criteria requiring early language onset abnormality, are in this account neither a primary consequence of cortical dysmaturation alone nor an incidental epiphenomenon of more diffuse neurodevelopmental disturbance. They are the patterned outcome of a developmental cascade. Prenatal deviation of cerebellar vermal lobules VI and VII, a phylogenetically recent neocerebellar region whose selective primate expansion is well documented, alters excitatory drive through cerebello-thalamo-cortical pathways during sensitive periods of postnatal cortical maturation. That altered drive contributes in turn to local cortical overconnectivity with reduced long-range integration, to posterior corpus callosum reduction, and to compromised cerebellar generation of forward predictive models. The scope of this claim should be stated without ambiguity. It is advanced for the language-delayed autistic disorder cohort in which the vermal and callosal findings were established, and it is not advanced as a claim about the full DSM-5 autism spectrum. Within that broader spectrum, the framework predicts that these signatures will be present only in the subgroup with retrospective early language onset delay and bidirectional vermal lobule VI and VII abnormality, and predicts their attenuation in samples pooled across the spectrum without that stratification. Convergent evidence supports this cascade across neonatal connectivity in relation to autism polygenic liability [214], thalamocortical alterations in six-week-old high-familial-risk infants [215], toddler motor-network connectivity [216], comparative primate prefrontal pathway disruption [218], and mTOR-dependent synaptopathy producing cortico-striatal hyperconnectivity in both Tsc2 mice and idiopathic autism [217]. The framework also specifies the cerebellum as the substrate for forward predictive models in auditory, social, and contextual learning, with recent computational accounts formalizing this role through Kalman-filter extensions to non-motor functions [53,235] and electrophysiological work documenting a graded dissociation in higher-order auditory predictive processing across autistic disorder and developmental receptive language disorder [84,85,86]. The account positions autism not only as a connectivity disorder but as a prediction disorder, with cerebellar vermal hypoplasia providing a specific neuroanatomical substrate for compromised predictive scaffolding during early development.

The framework has identifiable boundary conditions, and several findings could disconfirm or substantially constrain it. Because a disconfirmation criterion that appeals to the absence of cerebellar pathology is only as strong as the definition of that absence, the term is used here to mean the conjunction of four operationally specified conditions: vermal lobule VI and VII area within one standard deviation of a typically developing reference sample on structural imaging, no reduction in cerebellar gray matter on voxel-based analysis, cerebello-thalamo-cortical functional connectivity within the typical range, and intact performance on a cerebellar-dependent predictive-timing task. A case meeting all four while showing the cortical connectivity signature would count against the account; subtle cerebellar dysfunction cannot be invoked post hoc to preserve it. The hypoplasia/hyperplasia subgroup distinction in Courchesne et al. [21] implies that vermal lobule VI–VII size alone is not sufficient as a one-to-one biomarker, and the model would require modification if a substantial subset of individuals meeting DSM-IV criteria for autistic disorder showed neither pattern. The cross-condition literature reviewed earlier indicates that vermal hypoplasia is not sufficient for the autistic-disorder phenotype. Conditions with severe and anatomically unambiguous vermal hypoplasia, such as Joubert syndrome and Dandy–Walker malformation, produce the cluster of features defining ASD only in a minority of cases. Moreover, several syndromic causes of co-occurring ASD involve molecular mechanisms that affect cerebellar and cortical substrates simultaneously, among them FMR1 silencing in fragile X, MECP2 dysfunction in Rett, 7q11.23 deletion in Williams, and the 15q11-q13 imprinting defect in Prader–Willi. The refined account is therefore one in which vermal hypoplasia in the autistic-disorder phenotype serves as a developmental-window marker that an upstream perturbation reached a stage at which posterior cerebellar tissue was vulnerable, with the cluster of cortical and cerebellar features defining the phenotype emerging when the perturbation also affects the cortical context-integration substrates that cerebellar predictive scaffolding ordinarily supports. The cascade pathway specified in this manuscript is one route by which combined disruption can produce the phenotype; the temporal-coincidence framing accommodates alternative routes in which cerebellar and cortical disruption arise from a single upstream cause. Tripathy et al. [286] provide a concrete instance of such a route: mutations in MAST1 produce a syndrome combining mega-corpus-callosum, cerebellar hypoplasia, and cortical malformations in the absence of megalencephaly, so that a single molecular lesion yields simultaneous involvement of the three structures whose joint disruption the present framework identifies as characteristic of the autistic-disorder phenotype; the same report identifies de novo MAST1 substitutions in patients with autism and microcephaly, linking the gene directly to the autism phenotype as well as to the malformation syndrome. Cases of this kind are not counterexamples to the framework but instances of the alternative route it explicitly accommodates, and they are informative precisely because the callosal, cerebellar, and cortical findings co-occur without either structure being upstream of the other. The limited cross-site replication of ASD connectivity findings when motion and site effects are stringently controlled [25] is consistent with the case-mixing concern but also imposes a ceiling on the expected effect sizes for any single mechanistic account. Three demonstrations would directly disconfirm the account. The first would show individuals with isolated postnatal cerebellar vermal injury displaying the full autistic-disorder cortical connectivity signature in the absence of independent cortical pathology. The second would show idiopathic autism without any cerebellar pathology displaying the same cortical connectivity signature as the bidirectional-vermal subtype. The third would show that targeted prenatal cerebellar perturbations in non-human primates, sparing cortical substrates, fail to produce the cortical phenotype. One existing line of evidence bears on the first of these tests and warrants explicit engagement. Clausi et al. [287] directly compared individuals with autism and individuals with cerebellar neurodegenerative pathology on theory-of-mind measures alongside voxel-based morphometry, reporting converging mentalizing impairment across the two groups with posterior cerebellar Crus II implicated in both, and with the autism group showing this pattern in the absence of accompanying cerebral gray matter alteration. Cundari, Vestberg, Gustafsson, Gorcenco, and Rasmussen [288], reviewing cerebellar structure and function across ASD, attention-deficit/hyperactivity disorder, and spinocerebellar ataxia type 3, similarly concluded that morphological differences in distinct cerebellar subregions produce distinct behavioral symptoms through disruption of specific cerebro-cerebellar circuits, while noting that the imaging evidence remains insufficient to settle the cerebellum’s role in these conditions. These findings indicate that cerebellar compromise disturbs mentalizing irrespective of when in the lifespan it occurs, which is what a forward-model account of cerebellar contributions to social prediction requires. However, they do not establish that adult-onset cerebellar degeneration reproduces the autistic-disorder phenotype, because the early language-onset profile, the cortical connectivity signature, and the developmental time course that define that phenotype are not reported in the degenerative groups. The present framework accommodates both observations by distinguishing the cerebellum’s ongoing contribution to social prediction, which is age-independent, from the developmental cascade, which requires disruption during the prenatal and early postnatal window in which cortical connectivity is being established. The sharper form of the disconfirmation test is therefore whether acquired cerebellar injury reproduces the cortical connectivity signature and the developmental language profile, not whether it produces mentalizing difficulty. Five research priorities follow. (1) Infant and toddler studies combining cerebellar functional connectivity, event-related potential measures of auditory and contextual prediction error, and behavioral indices of socially contingent learning provide the most direct conjoint test of the cascade and predictive-modeling components. (2) Maintaining a principled distinction between autistic disorder and Asperger syndrome at the cerebellar and callosal level may help resolve apparent inconsistencies introduced by DSM-5 case-mixing. (3) A stratified re-analysis of large multisite samples, such as ABIDE I and II, by DSM-IV diagnostic subtype and retrospective language onset, testing for bidirectional vermal deviation rather than for volume reduction alone, represents a discrete and feasible empirical test of the case-mixing interpretation. (4) The contrast with developmental receptive language disorder offers a useful comparator for isolating cerebellar predictive contributions from generic auditory or developmental risk. (5) Transdiagnostic comparisons examining cerebellar and thalamic-cortical connectivity across autistic disorder, attention-deficit/hyperactivity disorder, and schizophrenia would test the generality of the account [183,185,191]. This last priority is tempered by the null result of Mizuno et al. [289], who found no significant differences in prefrontal cortex, cerebellum, or basal ganglia volumes between 92 children and adolescents with comorbid ASD and ADHD and 141 typically developing controls, the only significant difference falling in the left postcentral gyrus. That result admits two readings. It is what the case-mixing account developed here predicts, since a comorbid DSM-5-era sample is exactly the composition expected to attenuate an effect specific to the language-delayed autistic-disorder subgroup. However, it is equally consistent with there being no cerebellar volumetric effect to detect in comorbid presentations at all. The present framework should not claim the finding as support without the stratified re-analysis that would distinguish those two possibilities. Such a re-analysis would also constrain the specificity of the cerebellar finding to autistic disorder and help calibrate the developmental-window framing across disorders with distinct ages of onset. Integration with molecular and synaptic-level mechanisms, including mTOR-related synaptopathy and dentate nucleus dysfunction [135,217], may eventually permit biologically defined subtyping. The account presented here is offered not as a final theory but as a specifically structured hypothesis that organizes existing observations and points toward the experiments most likely to refine or refute it.

## 4. Conclusions

The present review has developed a mechanistic account of cerebral connectivity differences in autism, treated throughout as the prototypic case within the broader class of severe mental illness, grounded in five convergent claims. First, the connectivity findings reviewed span four decades during which the diagnostic criteria for autism themselves underwent substantial revision, and the foundational structural and connectivity findings were established in cohorts ascertained under DSM-III and DSM-IV criteria, under which ascertainment enriched samples for the autistic disorder subgroup with delayed language. Inconsistent replication of these findings in contemporary multisite cohorts, relative to those earlier samples, is consistent with diagnostic case-mixing as one contributor to attenuated group-level effects. It is one of at least four candidate contributors, alongside biological heterogeneity within the diagnosis, methodological and site-level variability, and the effect-size inflation characteristic of early small samples. The present evidence does not establish their relative magnitudes, and the case-mixing account is advanced as a testable competing explanation rather than as the established one. Second, deviation of cerebellar vermal lobules VI and VII from typical development, reported bidirectionally in autistic disorder samples and on evidence consistent with prenatal origin [21], is proposed to initiate a developmental cascade that shapes postnatal cerebral connectivity through cerebello-thalamo-cortical pathways during sensitive periods. The supporting evidence from imaging, postmortem, animal-model, and computational work is convergent, although a portion of the foundational human literature derives from overlapping research programs and partially overlapping cohorts, so convergence across reports should not be read throughout as fully independent replication. Third, the cerebellar contribution is most accurately framed as a developmental-window marker: the autistic-disorder cluster of features emerges when an upstream perturbation also affects cortical context-integration substrates at the relevant window, with cross-condition evidence (Joubert, Dandy–Walker, fragile X, Rett, Williams, Prader–Willi, schizophrenia) indicating that vermal abnormality alone is neither sufficient for the cluster nor specific to autism. Fourth, the cerebellum operates within this framework as the substrate for predictive internal models that scaffold auditory, social, and contextual learning, positioning autistic disorder as both a connectivity disorder and a prediction disorder. Fifth, the account generalizes beyond autism. Cerebellar involvement is transdiagnostic across severe mental illness, appearing in the psychotic spectrum, in obsessive–compulsive disorder, and in the genetic syndromes carrying elevated psychiatric risk, while the specific cerebellar territory implicated differs by condition. Autism is therefore the prototypic case rather than the exceptional one. It is where the developmental evidence is richest and where the cerebellar findings were first established, and it supplies the template against which the comparison conditions are read. The framework yields specific, testable predictions for infant and toddler studies and for stratified re-analysis of large multisite samples, and constrains future theorizing about diagnostic, mechanistic, and translational research in autism. However, this framework also suggests a cautionary consideration. The significant variability among individuals who may have many different forms and patterns of overlapping neurosubstrates, both anatomical and physiological, atypical patterns of connectivity, and an array of social, affective, cognitive, and behavioral manifestations that are likely attributable to such biological divergence and heterogeneity complicates the applied language label, e.g., the diagnosis. The term spectrum, as a noun, carries real advantages. It accommodates observed heterogeneity without imposing categorical boundaries that the evidence does not support; it has improved case identification and access to services; it acknowledges dimensional variation that the older categorical systems obscured; and it reflects the genuine difficulty of drawing defensible lines within continuous distributions of severity. However, its costs fall precisely where neurobiological research is conducted. The term is largely atheoretical relative to the very substrates and physiology that drive phenotypic expression, and a diagnostic label agnostic about mechanism will group together cases whose mechanisms differ. The consequence, documented throughout this review for autism and anticipated here for the other severe mental illnesses as their criteria broaden, is that biologically real effects present within a subgroup are attenuated when analyses pool across the spectrum, and that attenuation is then misread as failure to replicate. Two primary approaches are suggested going forward. First, small-scale studies with well-described cases involving social, affective, cognitive, and behavioral manifestations that probe the convergence of such domain-specific neurobiological substrates and physiology (an anti-spectrum approach). The second approach involves large-scale studies that have the capacity to sample targeted participants across the relevant diagnostic spectrum, but would also provide sufficient statistical power to detect multiple levels of anatomical substrate and physiology among participants who are much more heterogeneous in age, gender, and clinical presentation. This could include, for example, studying both the autism spectrum and the schizophrenia spectrum, and particularly those with early-onset schizophrenia.

Read at the level of the class, the value of the account lies in the fine detail rather than in the general claim. That cerebellar abnormality occurs across severe mental illness is by now unsurprising. What distinguishes these conditions from one another, and what may eventually define their neurobiology, is the more granular pattern: which lobules are affected, in which direction the deviation runs, at what developmental point the perturbation occurs, and which cortical systems are being organized when it does. Posterior vermal reduction with bidirectional deviation in the language-delayed autism phenotype, vermal enlargement in one schizophrenia sample alongside reduced total cerebellar volume in another and a dispersed lobular distribution in first-episode drug-naive psychosis, circuit-level cerebello-thalamo-cortical disruption without a vermal claim in obsessive–compulsive disorder, and anterior vermal reduction in fetal alcohol spectrum are not variations on a single finding. They are distinguishable signatures. Treating them as such is what converts a transdiagnostic observation into a differential neurobiology, and it is at this level of resolution that cerebellar findings become potentially biologically discriminating rather than merely transdiagnostically implicated.

## Figures and Tables

**Table 1:** Diagnostic criteria for autism across DSM editions, and the terminology used in this review to distinguish cohorts ascertained under each. The elimination of the language onset domain in DSM-5, together with the absorption of Asperger’s disorder into a single spectrum diagnosis, is the change with the largest consequence for sample composition in connectivity research.

DSM Edition (Year)	Early Language Onset Requirement	Autism-Related Categories	Term Used in This Review
DSM-III (1980)	Required; onset before 30 months	Infantile autism	Autistic disorder (DSM-III/IV cohort)
DSM-III-R (1987)	Not strictly required; polythetic list, eight of sixteen items across three domains	Autistic disorder	Autistic disorder (DSM-III/IV cohort)
DSM-IV (1994)	One of three qualifying onset domains; criterion for the autistic disorder versus Asperger’s distinction	Autistic disorder; Asperger’s disorder; PDD-NOS	Autistic disorder; Asperger syndrome reported separately
DSM-IV-TR (2000)	As in DSM-IV	As in DSM-IV	As in DSM-IV
DSM-5 (2013)	Eliminated	Single autism spectrum disorder diagnosis	Autism spectrum disorder (ASD)
DSM-5-TR (2022)	Eliminated	Single autism spectrum disorder diagnosis	Autism spectrum disorder (ASD)

**Table 2:** Cerebellar and connectivity findings across severe mental illness, with autistic disorder as the prototype case. The table is not offered as a comprehensive review of any condition other than autism. It provides the comparative context within which the autism findings should be evaluated. In cohorts ascertained under criteria requiring early language onset abnormality, the cerebellar finding localizes to vermal lobules VI and VII, deviates in both directions within the same sample, and is absent where language onset was normal. It attenuates once the language requirement is dropped. The comparison conditions implicate the cerebellum consistently but not at that locus. Schizophrenia shows enlargement at vermal VI and VII in one small single-site sample, reduced total cerebellar volume in a large multisite one, and, in first-episode drug-naive samples, a distributed pattern spanning lobules III to VI, VIII, and Crus I and II, rather than the focal posterior vermal reduction seen in autistic disorder. Obsessive–compulsive disorder implicates the cerebello-thalamo-cortical circuit without a vermal claim. The genetic syndromes distribute across the anterior vermis, posterior vermis, and cerebellar cortex. Cerebellar involvement is therefore transdiagnostic across severe mental illness, while the vermal lobule VI and VII signature is specific to the classically defined autism phenotype. Each study is identified using the diagnostic terminology employed by its own authors. The diagnostic frame column records the criteria in force at the time of ascertainment rather than a reclassification into current terms.

Study [Ref No.]	Method	Sample and Diagnostic Frame	Principal Finding	Cerebellar Abnormality	Connectivity Abnormality
**PROTOTYPE CASE. Autistic disorder: DSM-III and DSM-IV cohorts, enriched for early language onset abnormality**					
Courchesne, Yeung-Courchesne, Press, Hesselink, & Jernigan (1988) [20]	MRI, midsagittal area	18 autistic participants, 12 controls; DSM-III	Hypoplasia of vermal lobules VI and VII with lobules I to V spared	Yes: vermis VI–VII, reduced	Not assessed
Murakami, Courchesne, Press, Yeung-Courchesne, & Hesselink (1989) [22]	MRI, volumetric	Autistic participants and controls; DSM-III	Reduced cerebellar hemisphere volume accompanying the vermal finding	Yes: hemispheres, reduced	Not assessed
Courchesne, Lincoln, Yeung-Courchesne, Elmasian, & Grillon (1989) [186]	Auditory ERP	Nonretarded autism, receptive developmental language disorder, controls; DSM-III	Reduced P3b and Nc amplitudes shared across autism and receptive language disorder	Not assessed	Yes: electrophysiological
Lincoln, Courchesne, Harms, & Allen (1993) [84]	Auditory ERP, contextual probability	Autism, receptive developmental language disorder, controls; DSM-III-R	Impaired use of contextual probability, consistent with degraded predictive updating	Not assessed	Yes: electrophysiological
Courchesne, Saitoh, Yeung-Courchesne, Press, Lincoln, Haas, & Schreibman (1994) [21]	MRI, midsagittal area	50 autistic participants; DSM-III-R	Bidirectional deviation: approximately 86 percent hypoplastic, 12 percent hyperplastic at vermal VI–VII	Yes: vermis VI–VII, both directions	Not assessed
Egaas, Courchesne, & Saitoh (1995) [24]	MRI, corpus callosum morphometry	Autistic participants and controls; DSM-III-R	Reduced posterior corpus callosum area	Not assessed	Yes: structural, interhemispheric
Lincoln, Courchesne, Allen, Hanson, & Ene (1998) [33]	MRI, midsagittal area	Asperger syndrome, no early language delay; DSM-IV	Vermal lobules VI and VII preserved where early language onset was normal	No: vermis VI–VII intact	Not assessed
Levitt, Blanton, Capetillo-Cunliffe et al. (1999) [65]	MRI, vermal area	Children with autism; DSM-IV	Reduced posterior vermal area, with the analysis extended to lobules VIII–X	Yes: posterior vermis, reduced	Not assessed
Stanfield, McIntosh, Spencer, Philip, Gaur, & Lawrie (2008) [32]	Meta-analysis, structural MRI	Pooled autism samples; DSM-III to DSM-IV	Reduced cerebellar and vermal measures, moderated by IQ	Yes: vermis and total, reduced	Not assessed
**PROTOTYPE CASE, BROADENED ASCERTAINMENT. Autism spectrum disorder: DSM-IV-TR and DSM-5, no enrichment for early language onset abnormality**					
Tyszka, Kennedy, Paul, & Adolphs (2014) [27]	Resting-state fMRI	High-functioning ASD adults; DSM-IV-TR	Largely typical resting-state connectivity at the group level	Not assessed	No
Olivito, Lupo, Laghi et al. (2018) [30]	Resting-state fMRI, seed-based	ASD adults; DSM-IV-TR	Altered dentate nucleus connectivity with cortical social-brain regions	Yes: dentate nucleus	Yes: cerebello-cortical
Traut, Beggiato, Bourgeron et al. (2018) [26]	Meta-analysis plus ABIDE re-analysis	Pooled ASD; DSM-IV-TR and DSM-5	Cerebellar volume difference attenuated or undetectable once age and sex are modeled	No at group level	Not assessed
King, Prigge, King et al. (2019) [25]	Resting-state fMRI, ABIDE I	Multisite ASD; DSM-IV-TR and DSM-5	Limited cross-site reproducibility of functional connectivity findings	Not assessed	Equivocal
van der Heijden, Gill, & Sillitoe (2021) [187]	Review, developmental neurobiology	ASD	Cerebellar developmental abnormality framed as a driver rather than a correlate	Yes: developmental	Yes: cerebello-cortical
Wells et al. (2026) [162]	Meta-analysis	Pooled contemporary ASD; DSM-5	Cerebellar effect sizes attenuate as diagnostic breadth increases	Reduced effect size	Not assessed
**COMPARISON. Schizophrenia and the psychotic spectrum**					
J. J. Levitt, McCarley, Nestor et al. (1999) [173]	MRI, vermal area and volumetry	30 male patients; DSM-IV	Vermal lobules VI and VII larger than controls in patients without perinatal injury	Yes: vermis VI–VII, enlarged	Not assessed
Andreasen & Pierson (2008) [188]	Review	Schizophrenia	Cerebellar dysfunction argued central via cortico-cerebellar-thalamocortical disruption	Yes	Yes: CCTCC
Moberget, Doan, Alnæs et al. (2018) [189]	Multisite structural MRI mega-analysis	983 patients, 1349 controls; DSM-IV	Reduced total cerebellar volume with altered cerebello-cerebral structural covariance	Yes: total volume, reduced	Yes: structural covariance
Ding, Ou, Pan et al. (2019) [190]	Meta-analysis of VBM, ALFF, and FCS	783 first-episode drug-naive patients, 704 controls	Cerebellar abnormality at lobules III–VI, VIII, and Crus I–II before any medication exposure	Yes: distributed, not VI–VII specific	Yes: connectivity strength
Fu, Sui et al. (2020) [183]	Dynamic functional network connectivity	311 schizophrenia and controls; 569 ASD and controls	Increased cerebellar-subcortical and cerebellar-sensorimotor reconfiguration common to both	Yes	Yes: shared across conditions
Du et al. (2021) [184]	Multimodal structural and functional MRI	~600 schizophrenia, ~1000 ASD, ~1700 controls	Lower default mode and sensorimotor integration with reduced cerebellar gray matter in both, weaker in ASD	Yes: gray matter, reduced	Yes
Hwang, Kwak, Cho et al. (2021) [191]	Resting-state fMRI, thalamic seeds	Schizophrenia	Thalamic connectivity alterations paralleling those described in autism	Indirect, via CCTCC	Yes: thalamocortical
Fryer, Ferri, Roach et al. (2022) [192]	Resting-state fMRI, thalamic seeds	Psychosis risk syndrome and early illness schizophrenia	Thalamic dysconnectivity present in the risk syndrome, before frank illness onset	Indirect, via CCTCC	Yes: thalamocortical, prodromal
Feng, Zheng, Zou et al. (2022) [193]	Resting-state fMRI, cerebellar networks	First-episode schizophrenia	Altered functional connectivity of cerebellar networks at first episode	Yes	Yes: cerebellar networks
Abram, Hua, Nicholas et al. (2024) [194]	Resting-state fMRI, pontine seeds	Psychosis spectrum, including schizophrenia	Pons-to-cerebellum hypoconnectivity associated with sensory prediction and hallucinations	Yes: input pathway	Yes: pontocerebellar, prediction-linked
Wang, Guo, Cai et al. (2026) [195]	Resting-state fMRI	Schizophrenia	Cerebellar-basal ganglia dysconnectivity related to motivational deficits	Yes	Yes: cerebello-striatal
Diao et al. (2026) [185]	Transdiagnostic connectivity analysis	*n* = 2176 across ASD, ADHD, schizophrenia	Shared transdiagnostic connectivity abnormality implicating the cerebellum	Yes	Yes: shared
**COMPARISON. Obsessive–compulsive disorder**					
Sha, Edmiston, Versace et al. (2020) [196]	Resting-state fMRI, circuit-level	OCD	Functional disruption of cerebello-thalamo-cortical networks	Yes: circuit-level	Yes: CCTCC
Xu, Zheng, Guo et al. (2023) [197]	Structural and functional MRI with coupling analysis	Severe and moderate OCD	Structural and functional deficits and altered structure–function coupling, graded by severity	Yes	Yes
Shobeiri, Hosseini Shabanan, Haghshomar et al. (2024) [198]	Systematic review of diffusion tensor imaging	OCD	Cerebellar microstructural abnormality reported across diffusion studies	Yes: microstructural	Yes: white matter
Yan, Shan, Li et al. (2024) [199]	Longitudinal resting-state fMRI with gene expression analysis	Drug-naive OCD patients	Cerebellar functional connectivity changes over treatment with associated gene expression patterns	Yes	Yes: longitudinal
**COMPARISON. Genetic syndromes carrying elevated severe mental illness risk**					
Jernigan & Bellugi (1990) [180]; Schmitt et al. (2001) [181]	MRI morphometry	Williams syndrome	Vermal lobules VI and VII enlarged relative to controls	Yes: vermis VI–VII, enlarged	Not assessed
Sowell et al. (1996) [174]	MRI morphometry	Fetal alcohol spectrum	Reduced anterior vermal lobules I through V, posterior vermis spared	Yes: vermis I–V, reduced	Not assessed
Sefik et al. (2024) [177]	Structural MRI	3q29 deletion, 40- to 100-fold schizophrenia risk	Reduced cerebellar cortex volume with an anterior–posterior gradient	Yes: cortex, reduced	Not assessed

## Data Availability

No new data were created or analyzed in this study. Data sharing is not applicable to this article.
